# Epinecidin-1, an Antimicrobial Peptide Derived From Grouper (*Epinephelus coioides*): Pharmacological Activities and Applications

**DOI:** 10.3389/fmicb.2019.02631

**Published:** 2019-11-20

**Authors:** Pui Ying Chee, Morokot Mang, Ern Sher Lau, Loh Teng-Hern Tan, Ya-Wen He, Wai-Leng Lee, Priyia Pusparajah, Kok-Gan Chan, Learn-Han Lee, Bey-Hing Goh

**Affiliations:** ^1^Biofunctional Molecule Exploratory Research Group, School of Pharmacy, Monash University Malaysia, Subang Jaya, Malaysia; ^2^Novel Bacteria and Drug Discovery Research Group, Microbiome and Bioresource Research Strength, Jeffrey Cheah School of Medicine and Health Sciences, Monash University Malaysia, Subang Jaya, Malaysia; ^3^Institute of Biomedical and Pharmaceutical Sciences, Guangdong University of Technology, Guangzhou, China; ^4^State Key Laboratory of Microbial Metabolism, Joint International Research Laboratory of Metabolic and Developmental Sciences, School of Life Sciences and Biotechnology, Shanghai Jiao Tong University, Shanghai, China; ^5^School of Science, Monash University Malaysia, Subang Jaya, Malaysia; ^6^Medical Health and Translational Research Group, Jeffrey Cheah School of Medicine and Health Sciences, Monash University Malaysia, Subang Jaya, Malaysia; ^7^Division of Genetics and Molecular Biology, Faculty of Science, Institute of Biological Sciences, Kuala Lumpur, Malaysia; ^8^International Genome Centre, Jiangsu University, Zhenjiang, China; ^9^Health and Well-being Cluster, Global Asia in the 21st Century Platform, Monash University Malaysia, Subang Jaya, Malaysia; ^10^College of Pharmaceutical Sciences, Zhejiang University, Hangzhou, China

**Keywords:** epinecidin-1, fish-derived, antimicrobial peptide, *Epinephelus coioides*, aquaculture

## Abstract

Epinecidin-1 is an antimicrobial peptide derived from the orange-spotted grouper (*Epinephelus coioides*). The mature epinecidin-1 peptide is predicted to have an amphipathic α-helical structure and a non-helical hydrophilic domain at the C-terminal RRRH. The majority of work studying the potential pharmacological activities of epinecidin-1, utilize synthesized epinecidin-1 (Epi-1), which is made up of 21 amino acids, from the amino acid sequence of 22–42 residues of Epi-1—GFIFHIIKGLFHAGKMIHGLV. The synthetized Epi-1 peptide has been demonstrated to possess diverse pharmacological activities, including antimicrobial, immunomodulatory, anticancer, and wound healing properties. It has also been utilized in different clinical and agricultural fields, including topical applications in wound healing therapy as well as the enhancement of fish immunity in aquaculture. Hence, the present work aims to consolidate the current knowledge and findings on the characteristics and pharmacological properties of epinecidin-1 and its potential applications.

## Introduction

Marine antimicrobial peptide may be novel therapeutic agents, given that they are produced by marine organisms such as fish as a first line of defense against the various pathogens in the sea (Shabir et al., 2018). Many major classes of AMP have been derived from fish, such as defensins, cathelicidins, hepcidins, histone-derived peptides, and piscidins (Zhuang et al., 2017), making fish a good source to explore for AMPs. To date, there are 3,065 AMP listed in “The Antimicrobial Peptide Database” and among them 125 originated from fish (http://aps.unmc.edu/AP/main.php). The growing demand of the orange spotted grouper as a food source and the significant reduction in culture production due to pathogenic infection has prompted increased interest in epinecidin-1 in the hope of treating fish disease in the aquaculture industry (Pan et al., 2007). Given the similarities in primary or secondary structures of the AMPs, Epi-1 belongs to the piscidin family which is an evolutionarily conserved family of linear, amphipathic AMP which is unique to fish and is homologous to cecropins (Masso-Silva and Diamond, 2014).

**Figure 1:**
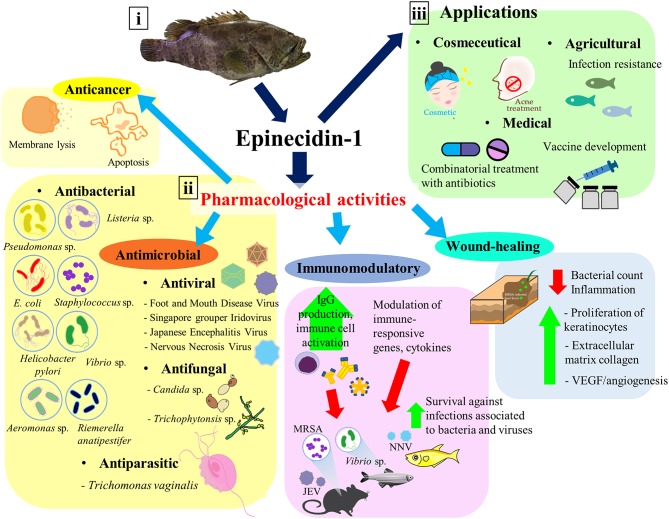
Epinecidin-1, an antimicrobial peptide with diverse pharmacological activities and applications. (i) The isolation source of epinecidin-1 cDNA—orange-spotted grouper *Epinephelus coioides*. (ii) Epi-1 has shown to exhibit antimicrobial, anticancer, immunomodulatory, and wound-healing activities. (iii) Due to its promising pharmacological activities, Epi-1 has potential applications in various areas, including medical, cosmeceutical, and agricultural industries.

To investigate the pharmacological activities of epinecidin-1, a synthetic Epi-1 peptide (21 amino acids) consisting of amino acid residues 22–42 from the amino acid sequence of epinecidin-1 has been successfully synthesized and purified for biological studies (Pan et al., 2007). Epi-1 peptide has been reported to exhibit broad spectrum antimicrobial activities, antiviral activity, immunomodulatory effect, and wound healing effect (Yin et al., 2006; Lin et al., 2009b; Pan et al., 2011; Huang et al., 2017b). This study reviews the structure and characteristics of epinecidin-1, the pharmacological activities of the Epi-1 peptide (Table 1) as well as its potential applications (Figure 1). To avoid confusion, the term “Epi-1” peptide in this review refers to the synthetic epinecidin-1 (amino acid residues 22–42), unless stated otherwise.

**Table 1:** Biological activities of Epi-1 peptide demonstrated in various *in vitro* and *in vivo* studies.

**Biological activities**	***In vitro*/*In vivo* studies**	**Findings**	**References**
Antimicrobial	MIC assay -MRSA	MIC−6.25 μg/mL	Huang et al., 2013
Antibacterial (Gram-positive bacteria)	MIC assay - MRSA strain isolated clinically from stool sample (Resistant to ampicillin, methicillin, oxacillin and ciprofloxacin)	MIC−9 μg/mL	Huang et al., 2017b
	Checkerboard titration method - Epi-1 is used to determine synergistic activity with clinically-used antibiotics (streptomycin or kanamycin) in inhibiting MRSA	Synergistic activity resulted in dramatic decreases in MIC for Ep-1 from 12.5 to 4.3 μg/mL against MRSA.	Lin et al., 2013
	*In vivo*: -A heat burned injury (3 cm) was created in pig model and treated with 10^10^ CFU/mL of MRSA (MRSA strain isolated clinically from stool sample which is resistant to ampicillin, methicillin, oxacillin, and ciprofloxacin). - At 6 h post infection with 10^10^ CFU/mL MRSA, the wounds were treated with Epi-1 concentrations equivalent to 0 (PBS only), 10 (90 μg/mL), 100 (900 μg/mL), 1,000 (9 mg/mL)-fold of the MIC for MRSA for 1 h. MRSA counts were estimated in surface wash sample concentrates	- The samples treated with the 1,000 (9 mg/mL)-fold MIC equivalent of Epi-1 showed no detectable MRSA counts - Epi-1 decrease MRSA levels in surfaces washes and superficial and deep halved of biopsies	Huang et al., 2017a
	MIC assay - *Staphylococcus aureus* (BCRC 10780)	MIC−12.5 μg/mL	Pan et al., 2007
		MIC−50 μg/mL	Lin et al., 2013
	MIC assay - *Staphylococcus* sp. (BCRC 10451)	MIC−50 μg/mL	Pan et al., 2007
		MIC−6.25 μg/mL	Lin et al., 2013
	-*Staphylococcus aureus sub*sp. (BCRC10782)	MIC−6.25 μg/mL	Pan et al., 2007
	MIC assay -*Staphylococcus agalactiae* (819)	MIC−50 μg/mL	Pan et al., 2007
		MIC > 21.41 μg/mL	Peng et al., 2012
	-*Staphylococcus agalactiae*	MIC > 100 μg/mL	Lin et al., 2013
	-*Staphylococcus agalactiae* (BCRC 10787)	MIC−0.33 μg/mL	Peng et al., 2012
	- *Staphylococcus epidermidis* (BCRC 10783)	MIC >100 μg/mL	Pan et al., 2007
	- *Staphylococcus xylosus* (BCRC12930)	MIC−50 μg/mL	Pan et al., 2007
	MIC assay - *Streptococcus pneumonia* (BCRC 10794)	MIC−25 μg/mL	Pan et al., 2007
		MIC−50 μg/mL	Lin et al., 2013
	MIC assay - *Streptococcus pyogenes* (BCRC 10797)	MIC−25 μg/mL	Pan et al., 2007
	MIC assay -*Propionibacterium acnes* (BCRC 10723)	MIC−200 μg/mL	Pan et al., 2009
	MBC assay -*Bacillus subtilis* (from soil)	MBC^a^ > 67.04 μM	Yin et al., 2006
	MIC assay -*Enterococcus faecalis* (BCRC 10066)	MIC > 21.41 μg/mL	Peng et al., 2012
	MIC assay -*Micrococcus luteus* (BCRC 11034)	MIC−25 μg/mL MIC−6.25 μg/mL	Pan et al., 2007 Lin et al., 2013
	MIC assay -*Listeria monocytogenes* (BCRC 10780)	MIC−50 μg/mL	Pan et al., 2007
Antibacterial (Gram- negative bacteria)	MIC assay - *Vibrio alginolyticus* (from dead grouper)	MIC−12.5 μg/mL	Pan et al., 2007 Lin et al., 2013
	- *Vibrio alginolyticus* (Grouper)	MIC−6.25 μg/mL	
	MBC assay - *Vibrio alginolyticus* (from *Epinephelus faria)*	MBC^a^-0.26 μM	Yin et al., 2006
	-*Vibrio alginolyticus* (BCRC12829)	MIC−12.5 μg/mL	Pan et al., 2007
	-*Vibrio alginolyticus*	MIC−2.68 μg/mL	Peng et al., 2012
	MIC assay - *Vibrio vulnificus* (204)	MIC−12.5 μg/mL	Pan et al., 2007
		MIC−50 μg/mL	Lin et al., 2013
		MIC−0.67 μg/mL	Peng et al., 2012
	MBC assay -*Vibrio vulnificus* (from culture water)	MBC^a^−4.19 μg/mL	Yin et al., 2006
	- *Vibrio vulnificus* (YJ016)	MIC−50 μg/mL	Pan et al., 2007
	MIC assay - *Vibrio harveyi* (BCRC 13812)	MIC−12.5 μg/mL	Pan et al., 2007
		MIC−6.25 μg/mL	Lin et al., 2013
	- *Vibrio harveyi* (BCRC 12907)	MIC−12.5 μg/mL	Pan et al., 2007
	MBC assay -*Vibrio parahaemolyticus* (from culture water)	MBC^a^ < 0.13 μM	Yin et al., 2006
	MIC and MBC assay - *Helicobacter pylori* (ATCC 43504, 700392, 51653 and antibiotic resistant clinical isolate	MIC−8–12 μg/mLMBC−12.5–25 μg/mL 2× and 1× MIC Epi-1 decreased *H. pylori* by 99% at 1- and 12-h post-exposure, respectivelyInteracts with the negative surface of *H. pylori*, and result in membrane disruption	Narayana et al., 2015
	MIC assay - *Enterobacter aerogenes* (BCRC 10370)	MIC−50 μg/mL	Pan et al., 2007
	- *Entereobacter cloacae* subsp. (BCRC 10401)	MIC−100 μg/mL	Pan et al., 2007
	MIC assay - *Klebsiella oxytoca* (BCRC13985)	MIC−100 μg/mL	Pan et al., 2007
	MIC assay - *Salinivibrio costicola* subsp. (BCRC 12910)	MIC−12.5 μg/mL	Pan et al., 2007
	MIC assay - *Pseudomonas aeruginosa* (ATCC 19660)	MIC−60 μg/mL	Pan et al., 2007
		MIC−10.70 μg/mL	Peng et al., 2012
		MIC_90_ of *P. aeruginosa* ATCC 19660–50 μg/mL imipenem−3.12 μg/mLMIC_90_ of *P. aeruginosa* R - 3.12 μg/mL imipenem - 200 μg/mL	Pan et al., 2014
	*In vivo* Mice infection model -*Pseudomonas aeruginosa* (ATCC 19660) - *Pseudomonas aeruginosa* R	- Untreated mice died within 72 h while Epi-1 decrease mortality rate. -7 days after infection, treatment with Epi-1 cause survival rate of 88.4% for both infections.	
	MBC assay - *Pseudomonas fluorescens* (from Culture water)	MBC^a^-67.04 μM	Yin et al., 2006
	MIC assay - *Yersinia enterocolitica* subsp (BCRC 13999)	MIC−100 μg/mL	Pan et al., 2007
	MIC assay - *Riemerella anatipestifer* (CFC 437, RA3, CFC27, RA16, T10,T6, A2, MRS)	MIC−25 μg/mL	Pan et al., 2010a
	MIC assay - *Riemerella anatipestifer* (CFC 363, RA9)	MIC−50 μg/mL	Pan et al., 2010a
	MIC assay - *Riemerella anatipestifer* (B2)	MIC−6.25 μg/mL	Pan et al., 2010a
	MBC assay -*Pasteurella multocida* (from *Lutjanus erythopterus)*	MBC^a^-0.26 μM	Yin et al., 2006
	MBC assay - *Morganella morganii* (from *Trionyx sinensis)*	MBC^a^-0.26 μM	Yin et al., 2006
	MIC assay - *Aeromanas hydrophila* (BCRC 13018)	MIC > 21.41 μg/mL	Peng et al., 2012
	MBC assay - *Aeromonas sobrio* (from *Ophiocphalus argus)*	MBC^a^-1.042 μM	Yin et al., 2006
	MBC assay - *Escherichia coli* DH5α (from Invitrogen)	MBC^a^-2.09 μM	Yin et al., 2006
	MBC assay -*Aeromonas hydrophila* (from Sparus latus *Houttuyn)*	MBC^a^-1.042 μM	Yin et al., 2006
	MIC assay - *Aeromonas hydrophila* BCRC 13018	MIC > 21.41 μg/mL	Peng et al., 2012
	MBC assay -*Flavobacterium meningosepticum* (from *Sinperca Chuatsi*)	MBC^a^ < 0.13 μM	Yin et al., 2006
Antiviral	JEV *In vitro*: Cell proliferation assay: Epi-1 was co-, pre-, and post-treated with JEV in BHK-21. Epi-1 concentration was from 0.0625 to 1 μg/ml. *In vivo*: (Mice) Viral challenge and Epi-1 treatments in mice: There were five groups: only Epi-1 (200 μg/ml), only JEV (50xthe LD50 of JEV;1.5 × 10∧7 pfu in 500 ml), JEV+Epi-1 (50,100,200 μg/ml). Rechallenged at day 14.	*In vitro*: Co-treatment of Epi-1 (at 0.5 and 1 μg/mL) shows 40 and 50% drop of JEV infection. Whereas, prophylactic and curative failed to prevent JEV infection *In vivo*: Co-injection of Epi-1 and JEV may induce some desirable adaptive immunity against JEV re-challenge. An ideal dosage against JEV infection in this given model system is 200 μg/mL of Epi-1.	Huang et al., 2011
	NNV & SGIV *In vitro*: TCID50 assay and RNA extraction: GS cells were treated with Epi-1, SGIV/NNV, and FBS to determine virus titer and analyze their gene expression	Epinecidin-1 (50 μg/mL) is effective reduce SGIV/NNV infectivity and inhibit the expression of SGIV ORF072 gene / NNV CP gene.	Wei et al., 2016
	NNV Antiviral activity assay: NNV (10^10^ TCID50 ml−1) was 100-fold diluted in L15 medium with or without 1% FBS, and the diluted NNV (10^8^ TCID50 ml−1) solutions were treated with Epi-1.	Epi-1 of 1,000 μg/mL was used in the assay. Results from log NI of Epi-1 was below 1.7 (with/without FBS). Also, free virions were observed in epinecidin-treated pNNV indicating that it could not block the infection nor agglutinate viral particles clump.	Chia et al., 2010
	NNV		Wang et al., 2010b
	*In vivo* (Grouper)		
	a. Co-treatment: 100,50, 10, and 5 μg/ml of Epi-1 with NNV b. Pretreatment: using Epi-1 (100 μg/ml) for PBS, NNV, and virus inoculated after 2, 4, and 8 h c. Post treatment: Epi-1 (100 μg/ml) in PBS, NNV, and after 8, 24, and 48 h NNV infection treatments d. Re-challenge e. RT-PCR analysis: The Mx2 and Mx3 genes were selected to represent interferon (IFN) response genes	a. Co-treatment: higher survival rate when treated with higher concentration of Epi-1. b. Pretreated in shorter amount of time shows higher survival rates. c. Post-treatment: earlier treatment shows higher survival rates d. re-challenge: there's an increase in survival rates e. The Mx2 and Mx3 genes were both downregulated in grouper treated with Epi-1	
	NNV		
	*In vivo* (Medaka)		Wang et al., 2010a
	a. Co-treatment: 100,50, 10, and 5 μg/mL of Epi-1 with NNV b. Pretreated: The pretreated groups were given a single injection of Epi-1 (100 μg/mL) and afterwards 2, 4, or 8 h, 10 μL RGNNV (10^8^ TCID50/ml) was injected. c. Post treated the post-treated groups were first injected with virus, and after 8, 24, 48, and 72 h, fish were given a single injection of Epi-1 (100 μg/mL); d. Re-challenge e. TEM and RT-PCR	a. Co-treatment: higher survival rate when treated with higher concentration of Epi-1. b. Pretreated in shorter amount of time shows higher survival rates. c. Post-treatment: earlier treatment shows higher survival rates d. Re-challenge: there's an increase in survival rates e. After Epi-1 treatment, the virus appeared to be surrounded by a sticky fluid with dark central color. Suggests that Epi-1 inhibits viral attachment to host cells by enhancing aggregation thus reducing NNV infectivity.	
	NNV *In vivo* (Medaka) Immunostaining and Immunohistochemistry: Medaka were infected with NNV and treated with or without Epi-1 then, tissue sections were taken from brain and eye ball. Control group: PBS Group1: NNV 10 μL (1 × 10^8^TCID50/mL) Group 2: 1 μl NNV +100 μg/mL Epi-1 Group 4: 10 μl NNV + 100 μg/mL Epi-1 (inject only after 8 h after infection)	Brain and eyes section of NNV-infected Medaka shows that co-treatment and post-treatment greatly decrease the stained from NNV antibody at 21 days compared to treated with NNV only.Immunohistochemistry: Small vacuoles were observed in the retinae and brain of medaka H12 infected with NNV, or treated with 1 μg of Epi-1 after 8 h post-infection	Wang et al., 2015
	FMDV *In vitro* MTS essay and a plaque reduction assay with BHK-21 cells	It has virucidal activity at 125 μg/mL and capable to interrupt with adsorption of FMDV on BHK-21 cells at 6.2 μg/mL	Huang et al., 2018b
Anti-parasitic	*In vitro*: *T. vaginalis* (ATCC 50143) (5 × 10^4^ cells/well) were cultured in a 96-well cell culture plate and treated with different concentrations of Epi-1 (7.8, 15.6, 31.2, 62.5, 125, 250, and 500 μg/ml) for 180 min or 1,440 min at 37°C. Samples were analyzed by microscopy and trypan blue exclusion. *In vivo* (Mice): AO staining and qPCR: Infected mice treated with 100, 200, 400 μg of Epi-1 daily for 7 days. Then vaginal punches were extracted and wet mounts were prepared to detect T. vaginalis and its 18S rRNA gene	*In vitro*:MIC - 62.5 μg/mL (membrane disruption and cell lysis). *In vivo*: Wet mount and AO staining showed that 400 μg of Epi-1 effectively eliminated T. vaginalis. At, 400 μg, it was calculated to have 92% cure rate toward *T. vaginalis* infection.	Huang et al., 2018a
	*In vitro:* Standard microdilution methods using 96- well microtiter cell. *T. vaginalis* (ATCC 30001, ATCC 50143 and T1)	MIC: *T. vaginalis* ATCC 30001: 12.5 μg/mL *T. vaginalis* ATCC 50143: 25 μg/mL *T. vaginalis* T1: 25 μg/mL	Pan et al., 2009
	*In vitro*: Standard microdilution method *T. vaginalis* (ATCC 30001, ATCC 50148, T1, MRS-1)	MIC:*T. vaginalis* ATCC 30001: 12.5 μg/mL*T. vaginalis* ATCC 50148: 25 μg/mL*T. vaginalis* T1: 25 μg/mL	Chen and Pan, 2010
Antifungal	*In vitro* - *C. albicans* SEM/TEM: Cells of *C. albicans* strain BCRC #20511 were cultured and treated with peptide Epi-1 at various concentrations.	MIC - 25 μg/mLSEM/TEM:shows plasma membrane damaged, irregular shape, and have shrunk compared to untreated *C. albicans*. Plus, observed to have seen plasma membrane damaged.	Pan et al., 2009; Chen and Pan, 2010
	*In vitro* - *C. albicans, Microsporosis canis, Trichophytonsis mentagrophytes, Cylindrocarpon* sp. Antimicrobial assay and hemolytic assay: Epi-1 (134 μM−0.033 μM) were mixed with an equal volume of microorganism suspension and incubated at 37°C for 60 min. Then spread on the respective agar plates and incubated for 18 h to determine the number of colonies formed	Yeast: - *C. albicans* MBC−8.38 μM Fungus: *Microsporosis canis*: MBC−16.76 μM *Trichophytonsis mentagrophytes*: MBC−33.52 μM *Cylindrocarpon* sp.: MBC−33.52 μM	Yin et al., 2006
Anti-sepsis	*In vivo* Duck infection models - *Riemerella anatipestifer* (strain MRS & T6) Duck model of aggressive bacterial infection was used with the RA strains of MRS and T6, major causes of duck septicemia. The concentration of *Riemerella anatipestifer* used (10^8^ cfu/duck) and	- Epi-1 significantly decreased mortality induced by the MRS strain in Cherry Valley ducks, and by the T6 strain in *C. moschata* ducks. Cherry ducks: (cotreatment) with 76.7% survival rates. Re-challenge at 14 days, the survival rates was 47.8%. - Epi-1 given 2 h prior shows 100% survival - Epi-1 given 2 h after challenge shows 20% survival.	Pan et al., 2010a
	Epi-1 (100 μg/duck) for pre, post, co-treatment and re-challenge.	*C. moschata*:Co-treatment has 100% survival rates - Rechallenge, 90% of the ducks survived. - Post treatment of Epi-1 has 100% survival rates pretreatment only shows 70% survival rates. - These results suggest that EPI-1 played significant roles in protecting ducks from RA-induced septic death	
	*In vivo:* - Mice infection model *P. aeruginosa* (Strain ATCC 19660 or R) Mice injected with *P. aeruginosa* ATCC 19660 or *P. aeruginosa* R and then injected with Epi-1 (0.005 mg/g mouse body weight) 10, 60, 120, 180, or 360 min later, survival rates recorded after 7 days	Epi-1 at 0.005 mg/g treatment in mice infected by ATCC 19660 and R was effective as curative agent when given within 10 to 120 min after infection.	Pan et al., 2014
Immunomodulatory effect	Zebrafish fed on transgenic *Artemia* expressing CMV-gfp-epi (*Vibrio vulnificus* (204) infection)	-Downregulation of TNF-α and MYD88 expression -Upregulation of defbl1, defbl2, defbl3, and hepcidin expression -Results show similar or lower survival rate in zebrafish fed on transgenic *Artemia* as compared to zebrafish fed on commercial fodder -Greatest increase in survival rate for zebrafish fed on commercial fodder combined with transgenic *Artemia*	Jheng et al., 2015
	Zebrafish injected with Epi-1 (*V. vulnificus* infection)	-Modulation of the expressions of immune-responsive genes like IL-10, IL-1β, TNF- α, and IFN-γ -Survival rate: - Pretreatment with 1 mg/ml of epinecidin-1 before injection of 10^3^ CFU of *V. vulnificus*: 56.6% - Post-treatment with 1 mg/ml of epinecidin-1 after injection of 10^3^ CFU of *V. vulnificus*: 60.0% -Co-treatment with 1 mg/ml of epinecidin-1 and 10^3^ CFU *V. vulnificus*: 78–97% after 30 days	Pan et al., 2011
	Nile tilapia fry fed on Epi-1-expressing *Artemia* cyst (*Streptococcus iniae*)	- Upregulation of IL-1β, IL-12, and CXCL-10 expression - Survival analysis showed that tilapia fry fed on Epi-1-expressing *Artemia* cyst were survived longer upon *S. iniae* infection when compared with the fry fed on control cysts. Tilapia fry fed on Epi-1-expressing *Artemia* cyst survived for 5 days upon infection as compared to control group fry which generally died within 3 days of infection	Ting et al., 2018
	Electrotransfer of the epinecidin-1 gene into skeletal muscle of grouper (*Epinephelus coioides*) (*V. vulnificus* infection)	With infection: - Downregulation of IL-1β and TNF expression -Prior electroporation with pCMV-gfpEpi showed greater inhibition of bacterial growth, as compared to electroporation with CMV-gfpWithout infection:	Lee et al., 2013
		-Upregulation of expression of MYD88, TNF-α, TNF2, NACHT, and IRF2	Lee et al., 2013
	Oral administration of the recombinant epinecidin-1 protein from BL21 Escherichia coli in grouper (*Epinephelus coioides*) and zebrafish (Danio rerio) for 30 days and 50 g of eel powder as fodder (*V. vulnificus* infection)	With infection: -Downregulation of TNF-1 expression -Significant increase in survival rate 13 days after infection after oral administration of the recombinant epinecidin-1 protein for 30 days	Pan et al., 2012
		-Higher dose was shown to give higher survival rates	
		Without infection:-Upregulation of expression of TNF-1 in grouper -Upregulation of Toll-like receptor (TLR)4, interleukin (IL)-1β, nitric oxide synthase (NOS)2, and nuclear factor (NF)-κB	Pan et al., 2012
	Injection of Epi-1 into mice (*P. aeruginosa* infection)	-Induce IgG1 production -Similar cell survival rate was shown in mice injected with *P. aeruginosa* and mice injected with both epinecidin-1 and *P. aeruginosa* after the primary infection	Lee et al., 2012
	Topical application of Epi-1 onto skin of mice (MRSA infection)	-Downregulation of TNF-a, IL-6, and chemokine MCP-1 -Epi-1 enhanced wound closure and angiogenesis as compared to vancomycin-treated infected mice -Gram staining observations revealed significant reduction in MRSA CFU as compared to control	Huang et al., 2013
	Mice injected intraperitoneally with Epi-1 (*P*. *aeruginosa* ATCC 19660 and *P. aeruginosa* R)	-Downregulation of IL-6, IL-1β, and TNF-α -Cotreatment with 0.005 mg/g epinecidin-1 decreased the mortality rate upon infection. - 7 days after *P. aeruginosa* ATCC 19660 and *P. aeruginosa* R infection, the survival rate is 88.4% - All untreated mice died within 72 h of infection.	Pan et al., 2014
	Transgenic zebrafish expressing Epi-1 was developed using an improved Tol2 transposon system (*Vibrio vulnificus* (204) and *Streptococcus agalactiae* infection)	-Significant upregulation of IL-22 and IL-26 expressions 12 h after a bacterial injection compared to wild-type zebrafish -No induction of immune-related molecule overexpression except for MyD88 before bacterial injection -Bacterial number gradually decreased from 6 to 24 h in the transgenic group	Peng et al., 2010
	Injection of Epi-1 into medaka (Nervous necrosis virus (NNV)	-Inhibit TGF-β1, TNF, BD, PVALB, CEBPA, IL-6ST, NF-κB2, and SP1 expression -Upregulation of certain genes involved in adipocyte signaling and B cell activation -Downregulation of expression of several genes involving in mast cell or T-cell activation	Wang et al., 2015
	Injection of Epi-1 into mice (JEV infection)	-Downregulation of IL-6, IL-12p70, MCP-1, TNF, and IFN-γ -Survival rates of mice re-challenged with 50 x the LD50 of JEV on the 35th day: - All Epi-1-JEV-immunized mice (n $\frac{1}{4}$ 13) survived - All but one of the formalin-JEV-immunized mice survived - All controls that died within 1 week.	Huang et al., 2011
Anticancer	*In vitro*: MTT cell viability, flow cytometry (PI staining), EtBr/AO staining ATP synthesis, DNA fragmentation (gel electrophoresis), Annexin V/PI flow cytometry, caspases-3,−8, and−9 assays Gene expressions	At 2–5 μg/mL, most effective at inhibiting U937 cellzVAD prevents proapoptotic activity of Epi-1 in U937, suggesting induction of caspase-dependent apoptosisIncreased ADP/ATP ratio, DNA latter formation, increased apoptotic cells, increased caspases-3, 8 and 9 activityUpregulated interleukin-related genes (IL-10)	Chen et al., 2009
	*In vitro*: MTT cell viability Soft gelatin cell colony formation test Scanning electron microscope Anti-necrosis properties, AO/EtBr straining, expression of necrosis-related genes	At 2.5 μg/mL, > 60% inhibition of cancer cells (A549, HA59T, HeLa, HT1080, U937) after 24 h incubationAt 2 μg/mL, > 90% inhibition of colony formation (A549 and HeLa)Act like lytic peptides, membrane disruption in HT1080 cells after 1-h incubationGranulation in cytoplasmic space of HT1080 cells, downregulation of necrosis-related genes (calpain 5 and cathepsin G), suggesting triggers anti-necrosis through cell membrane lytic effect	Lin et al., 2009b; Chen, 2012
Wound-healing	*In vitro*: Cell cycle analysis, Scratch assay *In vivo*: Heat-burned pig skin infected with MRSA Histochemical analysis (H&E, Giemsta, and Masson's trichrome staining)	- Increases S-phase cells, induces HaCaT keratinocyte cells proliferation to cover wounded region Heals the injury within 25 days after 1-h application at 6 h post-infection Enhances vascularization and epithelial activities Increase neutrophil recruitment to the injury site Enhances extracellular collagen formation	Huang et al., 2017a
	*In vivo*: Skin trauma-mediated MRSA infection in mice ELISA, histological, and immunohistological approaches	Reduces MRSA bacterial counts in wounded region, enhances wound closure, and increases angiogenesis at injury site, subsequent increased survival rate Decreases proinflammatory cytokines (TNF-α, IL-6 and MCP-1), Induces vascular endothelial growth factor (VEGF) Regulates monocytes recruitment and lymphocytes clearance	Huang et al., 2013
	*In vivo:* quantitative real-time RT-PCR	Induce the production of glial fibrillary acidic protein (GFAP)	Huang and Chen, 2013

a *Indicate that the synthetic (25 aa) epinecidin-1 mature peptide*.

*MIC, minimum inhibitory concentration; MBC, minimum bactericidal concentration; JEV, Japanese encephalitis virus; NNV, nervous necrosis virus; FMDV, foot mouth disease virus; SGIV, Singapore grouper Iridovirus*.

## Overview of Epinecidin-1

In 2006, epinecidin-1 was identified for the first time from an orange-spotted grouper leukocyte cDNA library. The length of the complete epinecidin-1 cDNA is 518 base pairs and consists of an open reading frame (204 base pairs) which encodes a sequence of 67 amino acids (Figure S1) (Yin et al., 2006). Based on the genomic information obtained from the peripheral blood of the grouper, the epinecidin-1 gene consists of four exons and three introns (Yin et al., 2006). Furthermore, the upstream sequence of epinecidin-1 gene was suggested to contain several putative regulatory elements and binding motifs for transcription factors, such as TATA box, binding sites for CAAT enhancer binding protein β (C/EBPβ), nuclear factor NF-κB, hepatocyte nuclear factors HNF-1 and HNF-3b (Yin et al., 2006). In the following years, epinecidin-1 was suggested to exist as a multigene family, similar to many other AMP genes that exist as multiple copies (Pan et al., 2008). In the study by (Pan et al., 2008), three epinecidin-1 genes have been identified in the genomic DNA library of the grouper. These three genes are denoted as Epi-1 124-1 (accession number: EU622517), 124-2 (accession number: EU622518) and 961 (accession number: EU622519). The results of the study revealed that both epinecidin-1 124-1 and 124-2 genes have a five-exon and four-intron structure, while the epinecidin-1 961 gene has a four-exon and three-intron structure (Pan et al., 2008). The study also showed that epinecidin-1 961 gene possesses 99% similarity to the gene sequence of epinecidin-1 gene (accession number: AY294407) identified from the study by (Yin et al., 2006). Furthermore, the study demonstrated the spliced exon sequences of the 124-1 and 961 genes resulted in translated amino acid sequences which are identical to those of translated epinecidin-1 cDNA (Pan et al., 2008). Moreover, several putative transcription factor binding sites, such as CdxA, SRY and AP-1 were also identified (Pan et al., 2008) besides those mentioned in the earlier study (Yin et al., 2006).

**Figure 2:**
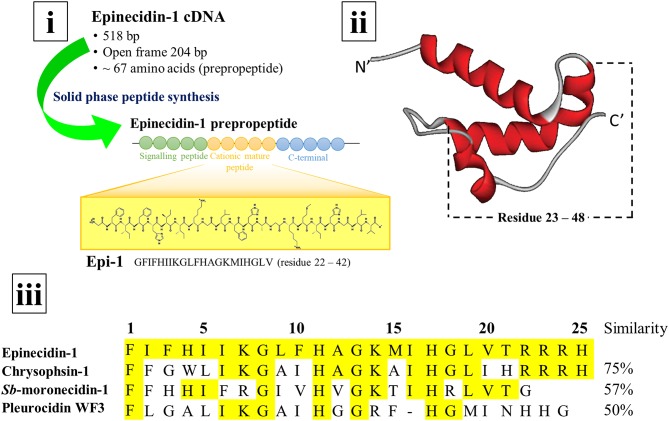
cDNA, secondary structure and amino acid sequence of epinecidin-1 (i) The cDNA of epinecidin-1 is isolated from the grouper *Epinephelus coioides* and the 21-mer epinecidin-1 (Epi-1) has been synthesized for biological activities study. (ii) The secondary structure of epinecidin-1 as an α-helix generated by Pan et al. (2007). (iii) The alignment of amino acid sequences between 25-mer epinecidin-1 with closely related piscidins computed from NCBI BLAST tool.

In general, AMPs exist as a long prepropeptide precursor encoded by the open reading frames in the genome. The 67 amino acid long epinecidin-1 prepropeptide consists of three domains: the first 22 amino acids constitute the hydrophobic signal peptide, the following 25 amino acids constitute the cationic mature peptide, and remaining 20 amino acids constitute the anionic C-terminal prodomain (Figure S1) (Yin et al., 2006). The N-terminal pre- or signal peptide usually functions in facilitating the translocation and secretion of the polypeptide during biosynthesis. Meanwhile, the C-terminal prodomain holds the AMP in an inactive form before further processing to be released when needed. The prepropeptide must undergo post-translational modification via proteolytic cleavage to release the active AMP. Thus, both signal peptide and prodomain of epinecidin-1 prepropeptide must be cleaved to release the biologically active mature epinecidin-1 peptide. Yin et al. (2006) determined that the mature epinecidin-1 consists of 25 amino acids with the sequence of FIFHIIKGLFHAGKMIHGLVTRRRH (amino acid residues 23-47). It has 9 basic amino acids: histidine (H), lysine (K), and arginine (R) which contribute to the net positive charge of +5. It has a molecular weight of 2985.63 Da and a pI value of 12.31. Furthermore, the anionic C-terminal prodomain is suggested to neutralize the cationic mature region before enzymatic cleavage (Yin et al., 2006). The epinecidin-1 peptide sequence was determined to be closely related to the peptide sequences belonging to the piscidins family: such as chrysophsin-1 from red sea bream, *Sb-*moronecidin-1 from hybrid sea bass and pleurocidin WF3 from winter flounder with sequence similarities of 78.6, 69.0, and 61.9%, respectively (Yin et al., 2006; Masso-Silva and Diamond, 2014). The secondary structure of epinecidin-1 peptide was predicted to have an amphipathic α-helical structure with hydrophobic and hydrophilic residues on opposite sides by using the Schiffer-Edmunson helical wheels modeling (Yin et al., 2006). Furthermore, Pan et al. (2007) constructed the structural model of epinecidin-1 peptide based on nuclear magnetic resonance studies and determined the structure of pleurocidin by homology modeling. Both epinecidin-1 peptide and pleurocidin were shown to possess a similar coiled pattern: both pleurocidin (amino acid residues 23–47) and epinecidin-1 peptide (amino acid residues 23–48) exist as an α-helical structure. Figure 2 depicts the secondary structure of epinecidin-1 and the alignment of amino acid sequences between mature epinecidin-1 peptide with closely related piscidins computed from NCBI BLAST tool.

Prior to 2018, there was no report on the direct isolation and purification of the mature form of epinecidin-1 peptide from *Epinephelus coioides (E. coioides)* grouper. Huang et al. (2018c) reported for the first time on the identification of a truncated peptide with 20 amino acids sequence (EGFIFHIIKGLFHAGKMIHG) which has 100% sequence similarity and 85.7% coverage to epinecidin-1 sequence (GFIFHIIKGLFHAGKMIHGLV). The truncated peptide (ecPis-1) was isolated from mucus of the grouper after challenge with *Vibrio parahaemolyticus (V. parahaemolyticus)*. The study also demonstrated that the ecPis-1 was successfully purified from the mucus but not from spleen and blood, suggesting the ecPis-1 was secreted from the mucosal immune system as the first line of defense against infection (Huang et al., 2018c). Moreover, the identification of the peptide in its truncated form in the study also supported previous proposals on the cleavage processing of the prepropeptide of epinecidin-1 to remove signal peptide and the prodomain. However, the processing of mature peptide of epinecidin-1 has yet to be completely elucidated. Thus, future research can focus on the identification of the enzymes and the cleavage sites involved in the post-translational modifications of the mature peptide of epinecidin-1.

In order to gain a deeper understanding of the importance of epinecidin-1 as a part of mucosal innate immunity, the expression of epinecidin-1 mRNA were investigated in the different organs involved in innate immunity. Epinecidin-1 mRNA is expressed in various adult skin tissues under normal conditions, including blood, gills, heart, head kidney, intestine, liver, spleen, stomach, brain, muscle, and skin. The expression of epinecidin-1 mRNA was higher in the head kidney, intestine, and skin, but significant increase of epinecidin-1 mRNA was detected in the head kidney 6 h after lipopolysaccharide (LPS) stimulation (Pan et al., 2007; Zhuang et al., 2017). The observation is supported by another study which reported the expression of Epi-1 transcript in head kidney, gills, skin, intestine was 2162, 607, 231, and 208 fold respectively of that in muscle under normal circumstances (Zhuang et al., 2017). The expression of epinecidin-1 mRNA on skin supported the hypothesis that epinecidin-1 plays an important role in innate immunity which is in accordance with the observation that epinecidin-1 mRNA was upregulated in the skin, gill, liver, kidney and spleen upon *Vibrio harveyi* JML1 challenge for up to 240 h post-infection indicating its importance in innate immunity (Pan et al., 2007; Amar et al., 2017). Stimulation *of E. coioides* using different doses of lipopolysaccharide (LPS) which is a component of outer membrane of Gram-negative bacteria and is associated with bacterial infection as well as polyinosinic-polycytidylic acid [poly(I); poly(C)] which is a synthetic analog of double-stranded RNA that acts as a molecular pattern associated with viral infection is done to study the LPS and poly(I);poly(C) regulation of epinecidin-1 expression (Alexander and Rietschel, 2001; Pan et al., 2007). The stimulation reveals that epinecidin-1 mRNA is expressed dose-dependently and helps in protecting *E. coioides* against viral and bacterial infection (Pan et al., 2007). Using immunohistochemical methods, epinecidin-1 peptide was shown to be expressed in the gills filamental epithelial cells and intestinal epithelial cells under normal conditions (Pan et al., 2007). However further research on the exact cell type involved in the production of Epi-1 is needed (Pan et al., 2007).

## Synthetic Epinecidin-1 Peptide (Epi-1)

As a large amount of raw biological sample is needed to obtain a fair amount of AMP and the purification of the AMP from nature sources is rather difficult due to post-translational processing, the chemical synthesis of peptides has become a preferred option (Haney et al., 2017). A 21 amino acid epinecidin-1 peptide—GFIFHIIKGLFHAGKMIHGLV (amino acid residues 22–42)—has been synthesized in majority of the studies on the biological activity investigation of the Epi-1 (Pan et al., 2007, 2010b, 2011; Lin et al., 2009b). Comparison to the deduced amino acid sequence of mature epinecidin-1 peptide in Yin et al. (2006), the synthetic 21-mer Epi-1 peptide has a sequence starting from residue 22 (glycine) instead of residue 23 (phenylalanine) compared to the longer 25-mer analog, FIFHIIKGLFHAGKMIHGLVTRRRH.

In a comparative study by Pan et al. (2007), the synthetic 21-mer Epi-1 peptide was shown to protect fish from infection but had less overall activity *in vitro* as compared to a longer epinecidin-1 30-mer peptide (GFIFHIIKGLFHAGKMIHGLVTRRRHGVEE). Despite that, the 21-mer Epi-1 was chosen by majority of the studies most likely due to the fact that the synthesis of a shorter peptide is more cost-effective (Yin et al., 2006; Pan et al., 2007; Huang et al., 2018c). The synthetic Epi-1 is generated via the solid-phase peptide synthesis method by using the Fmoc protecting group, this method is capable of generating a large quantity of the peptide at a reasonable price. The generated peptide is purified by reverse-phase high performance liquid chromatography (RP-HPLC) prior to verification for its identity and purity. The molecular mass and purity of the peptide is analyzed by mass spectrometry (MS) (Pan et al., 2007; Lin et al., 2013) to ensure peptide of high purity to be obtained (Haney et al., 2017). The 21-mer Epi-1 was also usually amidated at the C-terminal in various studies as many marine AMP has amidated C-terminal which helps to improve the antimicrobial activity (Yin et al., 2006; Pan et al., 2007; Chen et al., 2009). Amidated Epi-1 also possesses better LPS binding, thus allowing improved self-promoted uptake across outer membrane (Laurent, 1984; Pan et al., 2007).

## Pharmacological Activities of Epi-1

### Antimicrobial Activities

#### Antibacterial Activity

Both 21- and 25-mer Epi-1 have been extensively studied for their antibacterial activities. They have been reported to possess broad spectrum antibacterial activities against both Gram-negative and Gram-positive bacteria in various *in vitro* and *in vivo* preclinical studies (Yin et al., 2006; Pan et al., 2007). In the earliest study of Epi-1, a C-terminal amidated synthetic analog of the 25-mer mature epinecidin was shown to exhibit potent bactericidal activity (Yin et al., 2006). The results indicated that the 25-mer synthetic Epi-1 highly active against the Gram-negative bacterial strains with minimum bactericidal concentration (MBC) of <2 μM, including *Vibrio parahaemolyticus, Vibrio alginolyticus, Pasturella multocida, Morganella morganii, Aeromonas sobrio, Aeromanas hydrophila, Escherichia coli* DH5α, and *Flavobacterium meningosepticum*. Meanwhile, the 25-mer Epi-1 peptide is slightly less effective against *Vibrio vulnificus* and *Pseudomonas fluorescens* with MBC values measured at 4.19 and 67.04 μM, respectively. Epi-1 was found to be ineffective against the Gram-positive *Bacillus subtilis* (Yin et al., 2006).

Furthermore, 21-mer Epi-1 peptide was revealed to be effective against different strains of *Helicobacter pylori* with MIC and MBC values determined between 8 and 12 μg/mL and 12.5–25 μg/mL, respectively (Narayana et al., 2015). The study further demonstrated dose- and time- dependent bactericidal effects of Epi-1 peptide toward *Helicobacter pylori* ATCC 43504. At 2x and 1x MIC of Epi-1, *H. pylori* counts were reduced by 99% at 1 and 12 h post exposure, respectively (Narayana et al., 2015). Interestingly, the study also showed that Epi-1 peptide exhibits synergism against a multi-drug resistant (MDR) clinical strain of *H. pylori* when combined with antibiotics, including amoxicillin, metronidazole, and clarithromycin (Narayana et al., 2015). Another study by Pan et al. (2014) demonstrated the promising antibacterial activity of Epi-1 peptide against an antibiotic resistant bacterial strain, Epi-1 was shown to exhibit lower MIC_90_ value (3.12 μg/mL) than imipenem (200 μg/mL) against a MDR *Pseudomonas aeruginosa* R strain (Pan et al., 2014).

The antibacterial activity of Epi-1 has also been demonstrated in various *in vivo* studies where Epi-1 has been shown to confer protection toward infectious diseases. The administration of Epi-1 peptide promoted survival of the animals after challenge or re-challenge with bacterial pathogens, such as *V. vulnificus* (Pan et al., 2007, 2011; Lin et al., 2009a; Lee et al., 2013; Jheng et al., 2015). An *in vivo* study in 2007 showed that co-treatment of tilapia and grouper with 1 × 10^3^ CFU/fish of *V. vulnificus* (204) and 0.1 μg of Epi-1 peptide lowers the mortality significantly (30%) as compared to injection with bacteria only with mortality of 96% in tilapia and 100% in grouper after 7-day post infection. The increase in survival may be due to destruction of bacterial walls by Epi-1 peptide as verified by electron microscopy (Pan et al., 2007). Moreover, co-administration of 1 μg/mL Epi-1 peptide and *V. vulnificus* (10^3^ CFU/fish) into zebrafish resulted in better survival (80%) after a period of 7 days compared to pre- or post-treatment of Epi-1 peptide with survival rates of 56.6 and 60% respectively. Besides protecting fish from infection, Epi-1 peptide has also been shown to be a potential monotherapeutic agent for multi-drug resistant *H. pylori* infection in rodents. C3H/HeN mice infected with *H. pylori* experienced a 100% bacterial clearance after treatment with Epi-1 for 2 weeks (Narayana et al., 2015); while the standard PPI-Triple therapy was only able to reduce the bacterial counts by 2-orders of magnitude compared to control.

Epi-1 is also effective against multidrug resistant bacterial strains, such as methicillin-resistant *Staphylococcus aureus* (MRSA) (Huang et al., 2013). MRSA is a common cause of nosocomial infection, and MRSA infection is mainly treated with vancomycin. However, the decreased susceptibility due to the emergence of vancomycin-resistant strains, result in urgent need for alternative agents (Huang et al., 2013, 2017b; Choo and Chambers, 2016). Epi-1 peptide represents a promising alternative to vancomycin in the treatment of MRSA as Epi-1 peptide was shown to be more effective at treating MRSA infection than vancomycin (Huang et al., 2013). The results of an *in vivo* study indicated that application of Epi-1 peptide induced higher closure rate of wounds infected with MRSA in mice as compared to the infected mice treated with vancomycin. The enhanced healing effect of Epi-1 peptide was associated with its effective anti-MRSA activities, thereby Epi-1 peptide appears to be more efficient at reducing the MRSA bacterial count in the infected area as compared to vancomycin (Huang et al., 2013). Similar group of researchers also demonstrated that Epi-1 peptide effectively promotes healing of MRSA-infected heat burn injuries in swine (Huang et al., 2017a). The study showed that topical application of Epi-1 at 9 mg/mL completely healed the burn wound-infected with MRSA in swine after 24 days. Furthermore, Epi-1 was also shown to attenuate induction of sepsis associated with MRSA infection in swine as indicated by the suppressed levels of C-reactive protein and pro-inflammatory cytokine IL-6 in the circulatory system (Huang et al., 2017a).

Besides the attenuation of sepsis associated with MRSA, Epi-1 has also demonstrated antibacterial activity against *Riemerella anatipestifer* and confers protection to ducks against *R. anatipestifer* induced septic death (Pan et al., 2010a). *Riemerella anatipestifer* is a Gram-negative bacterium which infects ducks and causes high mortality, septicemia, and polyserositis. Given the MRS and T6 strains of *R. antipestifer* are the major causes of duck septicemia, these were used as bacterial infection in duck model. In the study by Pan et al. (2010a), pre-, post and co-treatments of Epi-1 have protective effect in duck model challenged by intraperitoneal injection of *R. anatipestifer* MRS strain (1 × 10^8^ CFU/duck) over 14-day experimental period. The co-treatment of Epi-1 (100 μg/duck) with MRS strain significantly decreased mortality of the Cherry Valley duck with survival rate of 77%, while only 23% survival rate was observed in control ducks treated with MRS strain. A significantly higher survival rate of 48% was also recorded for Cherry Valley ducks treated with Epi-1 after re-challenge with MRS strain as compared to the 17% survival rate recorded for the group receiving MRS strain only. It was hypothesized that the bacterial debris stimulates the immune response and thus protects against the re-challenge, indicating the potential usage of Epi-1 as both prophylaxis or therapy (Pan et al., 2010a). The protective effect of Epi-1 was also evaluated on different *R. anatipestifer* strain and duck models (*Cairina moschata*). The results showed that Epi-1 decreased mortality of *C. moschata* infected with T6 strain, whereby co-treatment, re-challenge, pre-treatment, and post-treatment resulted in 100, 90, 70, and 100% survival rates, respectively, compared to *R. anatipestifer* treated only had 30% survival rates. Furthermore, the study also indicated significant decrease in bacterial counts in duck liver after Epi-1 pre-, co-, and post-treatment. These results suggest that Epi-1 has strong antibacterial activities against *R. anatipestifer* and confers protection for duck from *R. anatipestifer*-induced septic death. Furthermore, it was demonstrated that Epi-1 was able to kill *Pseudomonas aeruginosa* R and ATCC19660 strains rapidly in mouse peritonitis sepsis model. Besides being able to enhance survival and decrease the bacterial numbers after the *Pseudomonas aeruginosa* peritonitis and sepsis in an infected mice model, Epi-1 was shown to exhibit curative effect when Epi-1 (0.005 mg/g) is immediately administered within 10–120 min of *Pseudomonas aeruginosa* ATCC 19660 and R stains infection with survival rates up to 93.3% (Pan et al., 2014). In addition to the evidence of Epi-1's effectiveness of saving animals from single species bacteria-induced sepsis, Epi-1 has also been tested against polymicrobial sepsis in mice induced by cecal-ligation puncture (CLP) surgery to simulate the diversity of infectious pathogens in human sepsis (Su et al., 2017). Intraperitoneal administration of Epi-1 was shown to successfully alleviate the inflammatory responses (reduced lung alveolar hemorrhage, immune cell accumulation, and systemic inflammatory markers), subsequently resulting in improved survival rate of C57BL/6 mice subjected to CLP surgery. A similar study also showed that Epi-1 inhibited LPS-induced endotoxemia in mice (Su et al., 2017).

To study the factors that affect the antibacterial activity of epinecidin-1, the activity of Epi-1 peptide against MRSA was examined under different circumstances. Irradiation with gamma rays (25kGy) or incubation of the Epi-1 peptide at high temperature reduces the antimicrobial activity against MRSA. Gamma irradiation may cause the formation of hydroxyl radicals by water radiolysis, thus degrading the peptide. Moreover, the peptide is not thermostable at temperatures higher than 40°C. It also favors a low pH level with the antimicrobial activity against MRSA being highest at pH2 and is lost at pH12. The high antimicrobial activity at a lower pH may be due to the amphipathic helix structural changes and dimers and transmembrane pores formation (Huang et al., 2017b). This observation is supported by the study done by Pan et al. (2010b) that shows C-terminal amidated Epi-1 is able to function at low temperatures (4°C) and claimed that epinecidin-1 is the first fish AMP that is active against pathogens at lower pHs, such as *H. pylori* which adapts the acidic environment in stomach. Good antimicrobial activity is also observed even after being dissolved in New Touch cleaning solution (Tien Liang Biotech, Taiwan) with pH ranges between 4.2 and 4.6. Thus, Epi-1 is able to function at a low temperature and pH environment.

Taken altogether, various *in vitro* studies have proven the broad spectrum bactericidal and bacteriostatic activities of Epi-1 against Gram-negative and Gram-positive bacteria. Epi-1 predominantly works by disrupting the membranes of the bacterial cells, as well as protecting the host through the intensification of cytoskeleton (Pan et al., 2007; Huang and Chen, 2013). Epi-1 can even act against multidrug resistant bacterial strains and poses lower chance of causing bacterial resistance compared to conventional antibiotics due to its direct antibacterial effect (Falanga et al., 2016; Zhang and Gallo, 2016). In addition, *in vivo* experiments also demonstrated promising antibacterial activities of Epi-1 in enhancing the survival of various host infection models (Pan et al., 2009, 2014; Jheng et al., 2015; Narayana et al., 2015; Huang et al., 2017b).

#### Antiviral

Numerous studies have demonstrated that Epi-1 possesses antiviral properties. Based on the literature, Epi-1 has been known to inhibit numerous types of viruses, including the Foot Mouth Disease Virus (FMDV), Nervous Necrosis Virus (NNV), Iridescent virus (Singapore grouper Iridovirus, SGIV), and Japanese encephalitis virus (JEV). According to Huang et al. (2018b), Epi-1 possesses antiviral activity against the FMDV (type O/Taw/97). The study further evaluated the anti-FMDV activity of Epi-1 after demonstrating that Epi-1 displayed the highest selectivity index (31.4) as compared to other synthesized AMPs with reference to protection of baby hamster kidney (BHK-21) cells against FMDV infection. Epi-1 was shown to exhibit virucidal activity against FMDV at a high concentration of 125 μg/mL (10 × EC_90_ of plaque assay) as demonstrated by the reduced FMDV infectivity; Epi-1 at a lower concentration (6.2 μg/mL) was capable of interrupting the absorption of FMDV onto BHK-21 cells. Thus, the study concluded that Epi-1 could interfere with the early stages of FMDV infection through mechanisms which are yet to be elucidated (Huang et al., 2018b).

Epi-1 also possesses antiviral activity against JEV. Through *in vitro* studies, Epi-1 at 0.5 or 1 μg/mL caused a drop of 40% and 50% of JEV infection respectively, when it was co-injected with JEV at multiplicity of infection (MOI) of 0.1 (equivalent to 5,000 pfu/well) in BHK-21 cells (Huang et al., 2011; Chen and Wu, 2013). Although prophylactic and curative treatment with Epi-1 failed to prevent JEV infection, it does play a role in JEV inactivation. On the other hand, *in vivo* studies involving co-injection of Epi-1 (200 μg/mL) and 50× the LD_50_ of JEV (1.5 × 10^7^ pfu in 500 ml) in mice suggested that Epi-1 may be able to induce adaptive immunity against JEV re-challenge. This was deduced as other test groups such as mice treated with JEV (and not co-injected with Epi-1) died within a week while the group that received Epi-1 only died after re-challenge. Thus, investigation on whether co-injection of Epi-1 and JEV can induce neutralizing antibodies production was performed. The results showed that co-injection of Epi-1 and JEV enhanced the production of immunoglobulin G1 (IgG_1_) which was increased further upon re-challenge, indicating that Epi-1 activates Th_2_ cells in response to JEV and produces IgG_1_ antibodies otherwise known as induction of humoral response. Epi-1 was also demonstrated to exert antiviral function by modulating expressions of immune-responsive genes and enhancing the pro-survival/antiapoptotic genes of host cells (Huang et al., 2011). Evidence of Epi-1 exhibiting anti-JEV activity *in vivo* exists as Epi-1 treatment reduced the multiplication of JEV in mouse brain. The findings were demonstrated by the detection of virus particles in the brain sections of adult mice treated with either JEV alone or JEV and Epi-1. The transmission electron microscopy (TEM) analysis showed that at the similar dosage of Epi-1 caused lysis of JEV (Huang et al., 2011; Chen and Wu, 2013).

The antiviral activity of Epi-1 against NNV, a betanodavirus has been studied more extensively than other viruses. NNV has been associated with mass mortality of aquaculture species, incurring immense economic loss in aquaculture industry. Thus, great attention has been given to AMPs with antiviral activity as good candidates for biocontrol agents of fishery-related viral infection. Epi-1 peptide was shown to be effective in reducing mortality of fishes infected with GNNV 9508 betanodavirus strain (Wang et al., 2010a,b). Pre-, co- and post-treatments of Epi-1 in grouper showed protective effects against NNV infection with significant reduction of mortality rate as compared to grouper infected with NNV only. Particularly, co-treatment of Epi-1 and NNV was shown to induce a > 2.60 fold increase in grouper survival as compared to those infected with NNV only (Wang et al., 2010b). The suggestion that Epi-1 had protective effects was also supported by the suppression of the NNV gene expression levels in grouper co-treated with Epi-1. Furthermore, Epi-1 was also shown to protect the grouper from repeated viral challenge and reduce mortality induced by NNV. Interestingly, both Mx2 and Mx3 genes, which are interferon response genes that function as innate defense against viral infection, were downregulated in groupers treated with Epi-1. The findings were unanticipated and suggested that Epi-1 may have induced other immune-related factors as well as exerting its antiviral function by reducing the infectivity of NNV (Wang et al., 2010b). Besides that, similar effectiveness in conferring protection toward NNV infection was also demonstrated with Epi-1 treatment in another fish model, medaka (*Oryzias latipes*) (Wang et al., 2010a). Co-treatment of NNV (at 1 × 10^6^ tissue culture infective dose (TCID)_50_/fish) and Epi-1 at highest concentration (1 μg/fish) produced > 72% survival rate as compared to NNV only. The survival rate was also enhanced even after re-challenge with NNV. Both pre- and post-treatments of Epi-1 also increased survival of medaka relative to medaka infected with NNV alone. The study further examined the effects of Epi-1 on the ultrastructure of NNV particles. The TEM results revealed that the viral particles were surrounded by sticky fluid with darker central color as compared to the PBS-treated virus. Treatment with Epi-1 was suggested to inhibit the spread of virus by inducing aggregation of viral particles into large and dense masses. Similar to response observed in grouper, co- and post-treatment of Epi-1 downregulated interferon gene expression in medaka. Thus, it was hypothesized that Epi-1 inhibits viral attachment to host cells by enhancing aggregation and encircling the virions at early stage of NNV infection, thus reducing NNV infectivity (Wang et al., 2010a).

To further investigate the antiviral mechanism of Epi-1 against NNV, transcriptome analysis was performed to examine the treatment effects of Epi-1 on the expression of immune genes in medaka infection model (Wang et al., 2015). The study revealed that there were 16 genes modulated by Epi-1 in response to NNV infection in medaka model. Based on the gene expression result, it was suggested that Epi-1 may have protected medaka against NNV by direct antiviral activity that destroys the virus as observed by the decreased immune gene expressions. Besides the evidence of reduced expression of NNV gene in response to Epi-1 treatment (Wang et al., 2010a,b), the viral loads in different organs of the infected medaka fish in response to Epi-1 treatment were investigated by immunohistochemistry using an NNV-specific antibody (Wang et al., 2015). The results showed absence of viral particles in brain and eye tissue at 7, 14, and 21 days post-injection when Epi-1 was co-injected with the virus. However, 8 h post-treatment showed the presence of some viral particles in retinae at day 7 that decreased over time. The study concluded that Epi-1 may either exert preventive action against NNV as observed by the decreased viral load when treated concurrently with Epi-1; while inducing host immune response to confer curative effect when Epi-1 is administrated after the infection. Furthermore, a patent has also been filed on antiviral activity of Epi-1 against NNV and SGIV (Wei et al., 2016). The patent demonstrated that grouper spleen (GS) cells pre-treated with Epi-1 resulted in reduced viral titer of SGIV and NNV. Besides, Epi-1 also inhibits the expression of SGIV ORF072 gene and NNV CP gene, suggesting the inhibitory effect of Epi-1 on the viral replication.

On the contrary, *in vitro* results showed that Epi-1 could not inhibit the infectivity of NNV on grouper fin-1 (GF-1) cell line (Chia et al., 2010). The study indicated that the pretreatment of Epi-1 (CP643-1) could not block the viral receptor of GF-1 cells to prevent entry of the virions into the cells. The TEM analysis further demonstrated that Epi-1 did not cause agglutination of virions as well as interfere with the viral adsorption and entry of cells. Intriguingly, Epi-1 was shown to induce Mx gene expression in cured barramundi brain (cBB) cell line. The study suggested that Epi-1 may confer protection against viral infection via modulating the immune genes in a host cell which was differed from another two AMPs investigated (Chia et al., 2010). Nonetheless, some of these contradictory findings suggest that more studies should be conducted to clarify the direct antiviral action of Epi-1 against NNV.

#### Anti-Parasitic

Anti-parasitic activity is another interesting property exhibited by Epi-1. A few studies have demonstrated the effects of Epi-1 against parasitic protozoan, *Trichomonas vaginalis* strain ATCC 30001, ATCC 50143, and T1 (the ATCC 50143 strain is metronidazole-resistant). *Trichomonas vaginalis* infection is a sexually transmitted disease which is also known as trichomonas or trichomoniasis. The symptoms of *T. vaginalis* infection include soreness, inflammation and itching, altered vaginal discharge and complications during pregnancy. It was indicated that treatment of Epi-1 induced morphological changes and death of T1 and ATCC 50134 cells (Pan et al., 2009). According to SEM and TEM analysis, disruption of plasma membrane, large amounts of cellular debris, and cell deaths were observed in the two strains of protozoans after 16 h of treatment with Epi-1. Moreover, through epifluorescence microscopy, *T. vaginalis* cells membrane were shown to be destroyed once treated with Epi-1. The results demonstrated that when *T. vaginalis* cells (4,000 cells/well) were co-treated with Epi-1 (200 μg/mL) for 16 h and then stained with cold PBS containing 1 μg/mL ethidium bromide (EtBr) and 1 μg/mL acridine orange (AO) for 10 min, red color was observed in the nuclei of *T. vaginalis* cells treated with Epi-1 suggesting that Epi-1 has lytic activities (Pan et al., 2009; Chen and Pan, 2010). To evaluate the effect of Epi-1 on the cell viability of ATCC 50143 and T1, treatments with Epi-1 at 25, 50, 100, or 200 μg/mL for 1, 4, and 16 h (overnight) were performed and followed by the analysis of cell viability using trypan blue exclusion assay. The results showed that Epi-1 exhibited lethal effect at 25 μg/mL, where rapid decrease in the number of the protozoans was observed within 1 h. Epi-1 was shown to kill the *T. vaginalis* cells in a time and dose-dependent manner (Pan et al., 2009; Chen and Pan, 2010). In addition, it was determined through propidium iodide staining and flow cytometric analysis that Epi-1 caused cell cycle arrest of T1 and ATCC 50143 strain cells (1 × 10^6^/mL) at G2/M phase and induced necrosis of cells (Pan et al., 2009). Another *in vitro* test indicated 62.5 μg/mL is the minimum inhibition concentration (MIC) of Epi-1 which causes 100% growth inhibition and cell lysis of *T. vaginalis* (Huang et al., 2018a). To investigate *in vivo* efficacy of Epi-1 against *T. vaginalis*, infected mice were treated with 100, 200, 400 μg of Epi-1 daily for 7 days. Then vaginal punches were extracted, and wet mounts were prepared for AO staining and qPCR to detect *T. vaginalis*. AO staining detected *e* while qPCR detected its 18S rRNA gene. Results showed 400 μg of Epi-1 effectively eliminated *T. vaginalis* which was comparable to furazolidone efficacy (Huang et al., 2018a). Clearly, Epi-1 has a high therapeutic index and is a product of natural selection in fish. Epi-1-containing pharmaceuticals can be formulated for systemic or topical administration. Overall, Epi-1 is a promising candidate for use in further clinical evaluation toward treating *T. vaginalis* infection.

#### Anti-Fungal

Epi-1 peptide also exhibits anti-fungal activities specifically against *Candida albicans* strain BCRC #20511 with an MIC of 25 μg/mL. *Candida* are a common cause of diseases such as atopic eczema, candida vaginosis, and septicemia. Based on the SEM and TEM results, significant differences were observed between the morphology of untreated *C. albicans* and *C. albicans* treated with Epi-1 at 50 μg/mL for 16 h. Disruption of plasma membrane, irregular shape, and shrinkage were observed in treated *C. albicans*, suggesting that Epi-1 exhibits promising anti-yeast activity (Pan et al., 2009; Chen and Pan, 2010). Besides having effects on yeast pathogens like *C. albicans*, Epi-1 was shown to be effective in killing numerous strains of fungus, including *Microsporosis canis, Trichophytonsis mentagrophytes*, and *Cylindrocarpon* sp. The minimum bactericidal concentration (MBC) of Epi-1 against *M. canis, T. mentagrophytes* and *Cylindrocarpon* sp. were determined at 16.76, 33.52, and 33.52 μM, respectively. Meanwhile, the MBC of Epi-1 (C-terminal amidated 25 amino acids residue 23-47) toward *C. albicans* was determined at 8.38 μM and MBC of more than 166 μM against *Pichia patoris* (Yin et al., 2006).

#### Immunomodulatory Effects

Epi-1 has also been demonstrated to exhibit immunomodulatory activities which confer protection against bacterial and viral infection in various animal models. The majority of studies examined the immunomodulatory effects of Epi-1 in diseased fish models using different application methods. A previous study examined the immunostimulatory effect of feed containing the recombinant epinecidin-1 peptide (expressed from BL21 *E. coli*) introduced into grouper and zebrafish orally. Without *V. vulnificus* infection, tumor necrosis factor alpha 1 (TNF-α1) was upregulated in grouper while Toll-like receptor 4 (TLR4), interleukin 1 beta (IL-1β), nitric oxide synthase 2 (NOS2), and nuclear factor (NF)-κB were enhanced in zebrafish fed with the recombinant epinecidin-1. Meanwhile, TNF-α1 expression was decreased in the fishes fed with the recombinant epinecidin-1 after challenge with *V. vulnificus*. The study suggested that dietary intake of recombinant epinecidin-1 regulated several immune-related genes, thereby conferring disease resistance in fishes (Pan et al., 2012). In addition, another study showed that Epi-1 peptide injected intraperitoneally into zebrafish modulated the expressions of immune-responsive genes like interleukin (IL)-10, IL-1β, TNF-α, and interferon-gamma (IFN-γ) as analyzed by a microarray and qPCR approach (Pan et al., 2011).

There were also studies evaluating the applications of *Artemia* as the vehicle for recombinant Epi-1 to fish for immunity enhancement. A study showed that zebrafish fed on commercial fodder combined with *Artemia* containing recombinant Epi-1 peptide for 7 days had lower expression of inflammatory genes such as tumor necrosis factor alpha (TNF-α) and Toll-like receptor (TLR) adaptor protein MyD88 in response to *V. vulnificus* (204) infection. Feeding zebrafish with similar combined diet for 7, 14, or 21 days also increased the expression antimicrobial peptide genes, defensin beta-like 1 (defbl1), defbl2, defbl3, and hepcidin after infection, suggesting the potential of Epi-1 to be a promising therapeutic agent for *V. vulnificus* (204) infection (Jheng et al., 2015). Another study revealed that Tilapia fry fed on epinecidin-1-expressing *Artemia* cyst resulted in the upregulation of IL-1β, IL-8, IL-12, and IFN-γ-inducible protein 10 (CXCL-10) expression, which is mediated by TLR-2/TLR-5 immune response (Ting et al., 2018). On top of that, electrotransfer of the epinecidin-1 gene into skeletal muscle of grouper increased the expressions of MyD88, TNF-α, TNF-α2, NACHT, and IRF2 (at varying times) without *V. vulnificus* infection. This suggested epinecidin-1 plasmid enhanced several immune-related genes to activate innate immunity in grouper. Meanwhile, upon infection, the expression of IL-1β and TNF were decreased with epinecidin-1 plasmid electroporation, supporting the protective role of Epi-1 against mortality caused by overexpression of immune-related genes (Lee et al., 2013). On the other hand, IL-22 and IL-26 expressions were shown to be higher in transgenic epinecidin-1 zebrafish 12 h after bacterial infection, suggesting that both IL-22 and IL-26 may be the key immune mediators released by transgenic zebrafish muscle (Peng et al., 2010).

Besides the evidence on immunomodulatory activities in fishes, Epi-1 has also been demonstrated to modulate different immune-related genes in mammals as well. Epi-1 was shown to induce an increase in IgG_1_ production via activation of Th2 cells in mouse model infected with *Pseudomonas aeruginosa* (Lee et al., 2012). Furthermore, inflammatory mediators such as IL-6, IL-1β, and TNF-α were also found to be greatly suppressed by Epi-1 in mice infected with *P. aeruginosa* ATCC 19660 (Pan et al., 2014). Similarly, Epi-1 also downregulated pro-inflammatory cytokines, including TNF-α, IL-6, and the monocyte chemoattractant protein-1 (MCP-1) in a skin trauma-mediated MRSA infection mouse model (Huang et al., 2013).

For the immunomodulatory effect of Epi-1 against viral infection, studies demonstrated that Epi-1 also confers protection to fish and mice infected by viruses via immune modulation. In the nervous necrosis virus (NNV) infected medaka fish, Epi-1 treatment was reported to inhibit expression of TGF-β1, TNF, BD, PVALB, CEBPA, IL-6ST, NF-κB2, and SP1. Epi-1 also induced expression of certain genes involved in adipocytokine signaling and B cell activation besides decreasing several genes involved in mast cell or T-cell activation (Wang et al., 2015). Epi-1 also downregulated proinflammatory cytokines such as IL-6, IL-12p70, MCP-1, TNF, and IFN-γ in mice infected by JEV (Huang et al., 2011).

Furthermore, an *in vitro* study was conducted to evaluate the immunomodulatory effect of Epi-1 on LPS-induced inflammation in macrophages. Epi-1 was demonstrated to inhibit cytokine production through suppression of LPS-induced activation of reactive oxygen species/p38/Akt/NF-κB signaling. The suppression was mediated by the ability of Epi-1 to disrupt the interaction between LPS and LPS binding protein (LBP) which inhibits Toll-like receptor 4 endocytosis (Su et al., 2017). To further decipher the role of Epi-1 in suppressing LPS/TLR-4 internalization, Su and Chen (2017) indicated that Epi-1 induced the degradation of TLR signaling adaptor protein, MyD88 via the activation of Smurf E3 ligase proteasome degradation pathway in Raw264.7 mouse macrophage cells.

#### Anticancer

Epi-1 has been also evaluated for anticancer activities. A previous study has reported that Epi-1 could suppress the proliferation of U937 cancer cells via apoptosis in a dose-dependent manner. The increase in ADP/ATP ratio due to mitochondrial damage after the cells were treated with 3 μg/mL of Epi-1 within 24 h suggested that apoptosis induced by Epi-1 was dependent on the mitochondrial membrane integrity. The activation of caspases-3,−8, and−9 within 4 h of treatment further reflected the involvement of mitochondria in apoptosis, as mitochondrial dysfunction could activate caspase-9, and then induces apoptosis by caspase-3. Furthermore, Epi-1 also upregulated interleukin-related gene, IL-10 in U937 cancer cells, suggesting that Epi-1 may also exert anticancer effect via immune modulation (Chen et al., 2009). Epi-1 was demonstrated to induce significant apoptosis of cancer cells at a concentration of 2.5 μg/mL but to a lesser extent on normal cells. According to soft gelatin cell colony formation test, Epi-1 was found to inhibit more than 90% of growth in A549, HeLa, and HT1080 cell lines (Chen, 2012). There was another study indicating that Epi-1 exhibits anticancer effect similar to lytic peptides. Epi-1 was suggested to cause cell membrane lysis with the evidence of orange color observed in HT1080 cells after AO/EtBr staining and DNA fragmentation analysis (Lin et al., 2009b).

#### Wound Healing

Epinecidin-1 has been shown to have wound healing properties. This role has gained more importance as wounds may become infected with resistant bacteria like methicillin-resistant *Staphylococcus aureus* which is one of the causes of invasive infection in burn patients which may bring about morbidity and even death (Norbury et al., 2016). A recent study reported that Epi-1 can heal MRSA-infected heat burn injuries by increasing the proliferation of keratinocyte cells, speeding up the formation of epithelial layer, enhancing the recruitment of neutrophils to the injury site and forming extracellular matrix collagen around the wound region. The wound was completely healed within 25 days with Epi-1 treatment for 1 h at 6 h post-infection (Huang et al., 2017a). This is further supported by another study which provided results that Epi-1 accelerated wound closure and enhanced angiogenesis at the wound region. This effect may be due to VEGF induced in Epi-1 treated tissues. The recovery was found to be further enhanced when combined with collagen (Huang et al., 2013). In addition, there is a possibility that Epi-1 is involved in the repair of neurological injury as Epi-1 was found to induce the production of glial fibrillary acidic protein (GFAP), a serum marker of traumatic brain injury found after central nervous system cell damage (Lumpkins et al., 2008).

#### Mode of Actions of Epi-1

**Figure 3:**
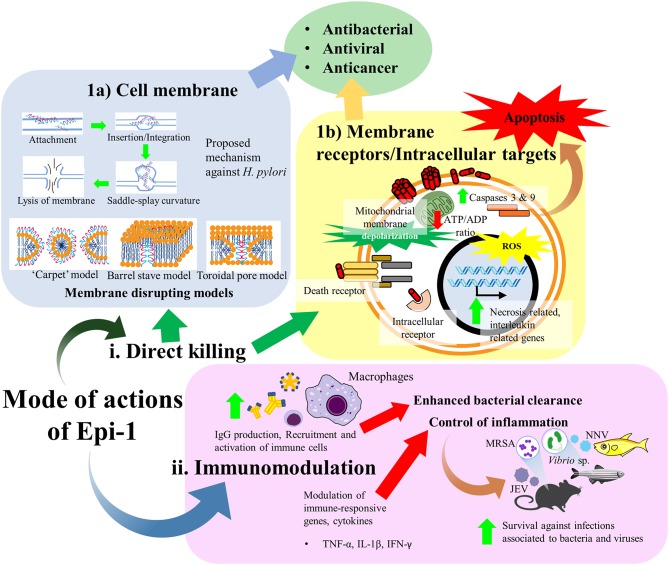
Mode of actions of Epi-1. Predominantly, the mode of actions of Epi-1 can be divided into (i) direct killing and (ii) immune modulation. The direct killing effect of Epi-1 is described to disrupt the membrane integrity of bacterial and cancer cells while there is also notion that Epi-1 may interact with receptors or accumulate intracellularly to interfere critical cellular processes. (1a) A proposed mode of action of Epi-1 against *H. pylori*. Epi-1 is initially attracted onto membrane of target cell prior to insertion or integration into the membrane leaflets. A nonzero curvature tension is created between the lipid molecules as a consequence of the insertion, hence inducing a saddle-splay curvature and leading to membrane vesicular budding or blebbing. Eventually, the membrane integrity is destabilized due to the extensive nonzero curvature tension, resulting the release of cellular contents (Narayana et al., 2015). Several models of membrane disrupting mechanisms were also suggested for the antimicrobial activity of Epi-1, including the “carpet,” barrel stave and toroidal pore models. (1b) Epi-1 also interacts with both outer membrane proteins and intracellular proteins on many different target cell types, including immune cells, keratinocytes, and cancer cells. Epi-1 binds with intracellular receptors or directly with membrane receptors to stimulate a variety of signal transduction pathways. (ii) Epi-1 regulates the immune-related genes and modulates the production of pro-inflammatory and anti-inflammatory cytokines to confer protection and disease resistance in host.

Epi-1 has been shown to exhibit a broad spectrum of bioactivities, including antimicrobial, anticancer, immunomodulatory, and wound healing activities. Nonetheless, the mechanisms underpinning the multifunctional activities of Epi-1 are, as yet, not fully understood. At the early stage, AMPs were considered molecules that “punch holes” in bacterial membranes. With the increasing research on AMPs, it has become apparent that their mechanism of action is much more complex and diverse. Studies show that these peptides vary from membrane permeabilization to actions on diverse extracellular and intracellular target molecules which in turn exert the corresponding bioactivities. Furthermore, AMPs play a crucial part in the host immune system. For instance, epinecidin-1 also demonstrates its importance as the innate immunity and acting as the first barrier of defense against pathogens. As evidenced, the epinecidin-1 mRNA is highly expressed within the innate immunity related organs of a host upon exposure to bacterial and viral infections (Pan et al., 2007; Zhuang et al., 2017). Thus, it is supposed that Epi-1 participates as an immunomodulators which can influence diverse ranges of immune responses and confer protections against bacterial and viral infections. Basically, the mode of actions of Epi-1 can be divided into direct killing and immune modulation. The direct killing effect of Epi-1 is predominantly described to disrupt the membrane integrity of bacterial and cancer cells while there is also notion that Epi-1 may interact with receptors or accumulate intracellularly to interfere critical cellular processes (Figure 3).

Generally, the antibacterial action of an AMP is attributed to the electrostatic attraction of the net cationic charge of AMP toward the negatively charged LPS on the outer membrane of Gram-negative bacteria or the lipoteichoic acids from the cell wall of Gram-positive bacteria causing non-enzymatic membrane disruption (Haney et al., 2017). Similarly, due to its cationic nature, Epi-1 could perhaps interact preferentially with the anionic lipids in the bacterial cytoplasmic membrane including phosphatidylglycerol and cardiolipin, causing membrane disturbance by displacing divalent cations (Fjell et al., 2012; Haney et al., 2017). Furthermore, as a linear AMP, Epi-1 can fold into an amphipathic structure in the hydrophobic environment of the cell membrane leading to multiple disruptions of the cell membrane (Huang et al., 2010). That being so, the selectivity of AMP depends on the membrane compositions of different microbes (Zhang and Gallo, 2016) as reflected by the differential antibacterial activities of Epi-1. Besides that, Epi-1 may also elicit anticancer activity by interacting with the membranes of cancer cells. As evidenced by previous studies, Epi-1 demonstrates selectivity of Epi-1 in killing cancer cells over healthy mammalian cells (Chen et al., 2009; Lin et al., 2009b). Although the detailed mechanisms of Epi-1 to select for cancer cells are lacking, it is generally accepted that this selectivity is dependent upon the fundamental differences between the cell membrane composition of normal and cancer cells (Tan et al., 2017). As compared to the overall neutral charge of normal cells, the net negative surface charge carried by cancer cells renders the targeting and electrostatic binding of cancer cells by the cationic Epi-1, suggesting that this could be a key determinant for the anticancer action of Epi-1 (Harris et al., 2013).

Over the years, various membrane disrupting models have been proposed such as the ATP dependent barrel-stave model, carpet model, and toroidal pore model as well as macropinocytosis which is ATP independent (Shabir et al., 2018). In fact, several different mechanisms had been proposed based on the structure of synthetic amidated epinecidin-1 putative mature peptide. One was the barrel stave model which involves the amphipathic α-helices inserting into the lipophilic center of the membrane and creating a transmembrane pore. The other mechanism proposed was the carpet model which involves the attachment of the peptides to the phospholipid head groups of target membrane without inserting into the hydrophobic core, inducing membrane disintegration by interrupting the membrane curvature and occuring when a threshold concentration is achieved (Shai, 1999; Yin et al., 2006). Figure 3 illustrates the carpet, barrel-stave and toroidal pore models as the proposed modes of action of Epi-1. The membrane disrupting effect of Epi-1 was shown by the increased uptake of fluorescence and the increase of positive charge when tested with 1-N phenylnapthylamine (NPN)-uptake assay and zeta-potential measurement of Epi-1 treated *Helicobacter pylori*. Furthermore, in the same study, TEM revealed that Epi-1 treated *H. pylori* appeared as ghost cells and was proposed that Epi-1 selectively induced saddle-splay membrane curvature (negative Gaussian curvature) which is important for processes such as pore formation, blebbing, budding and vesicularization to disrupt and lyse the membrane and the cell content leak out through the introduced pores leading to cell death (Narayana et al., 2015). Figure 3 depicts the proposed mechanism of Epi-1 against *H. pylori*. Besides that, TEM and scanning electron microscopy (SEM) revealed that the membranes of Gram positive *Propionibacterium acnes* were also disrupted and intracellular inclusions were effluxed extracellularly after 100 μg/mL Epi-1 treatment, similar TEM result was obtained for *R. anatipestifer* (RA) strain of MRS, CFC27, and T6 after 100 μg/mL Epi-1 treatment over 16 h (Pan et al., 2009, 2010a). Membrane lysis was also noted in Epi-1 treated *P. aeruginosa* cells under TEM (Pan et al., 2014). As for the antiviral mechanism, previous studies characterized the direct anti-viral properties of Epi-1 in terms of its inhibition of viral multiplication, adsorption, and infectivity. Despite that, the exact antiviral mechanism of Epi-1 is not clearly understood.

Although several mechanism of actions of Epi-1 have been proposed, it should be noted that a specific type of membrane destabilization mechanism may not represent the overall membrane mechanism of activity for an AMP (Schmidt and Wong, 2013). Moving away from the membrane destabilization, Epi-1 is believed to manifest its multitude pharmacological activities via interactions with both outer membrane proteins and intracellular proteins on many different target cell types, including immune cells, keratinocytes, and cancer cells. To achieve these, such AMP is generally capable to freely translocate across plasma membrane to bind with intracellular receptors or directly with membrane receptors to stimulate a variety of signal transduction pathways. For instance, besides the lytic effect of Epi-1 on cancer cells, Epi-1 was suggested to disrupt the mitochondrial membrane integrity, leading to mitochondrial dysfunction, depletion of ATP, and eventually apoptosis of cancer cell. Furthermore, Epi-1 was suggested to bind with death receptors and trigger the death receptor signal transduction pathway in U937 cancer cells (Chen et al., 2009). There is also evidence of Epi-1 modulating different genes in cancer cells, including necrosis related genes (calpain 5 and cathepsin G) and interleukin related genes (Chen et al., 2009; Lin et al., 2009b).

In addition to direct killing of pathogens, Epi-1 has been shown to regulate the immune-related genes and modulate the production of proinflammatory cytokines to confer protection and disease resistance in host (for detailed descriptions please refer to section “4.3. Immunomodulatory effect”). Basically, these evidences indicate that Epi-1 can bolster the immune response by modulating immune cell signaling and recruitment to enhance pathogen killing or control of inflammation (Lee et al., 2012). Furthermore, there is evidence that Epi-1 is also involved in modulation of the adaptive immune system, such as the Th2 and B cells (IgG1 production) in response to bacterial and viral infections (Huang et al., 2011; Lee et al., 2012). Despite the exact mechanism of Epi-1 remaining inconclusive, Epi-1 evidently acts by direct disruption of bacterial membranes and immune modulation. The membrane disruptive mechanism represents an important aspect for Epi-1, whereby it reduces the chance of bacterial resistance due to the fact that development of resistance requires change in bacterial membrane (Falanga et al., 2016; Huang et al., 2017b). Nonetheless, future studies should emphasize on the exploration of the structure-function and mechanism of Epi-1 to fully map out its immunomodulatory abilities in disease. With the advancement in bioinformatics and proteomics, the Epi-1 induced host defense mechanism should be more comprehensively studied and elucidated. As revealed by a proteomic study, Epi-1 induces significant changes in the cellular assembly and organization, and cellular function and maintenance, such as by regulating ubiquitination which is a form of protein modification for regulation of most cellular processes. Strengthening the cytoskeleton network is crucial to act as a barrier to protect the organism from microbial challenges (Geisler and Leube, 2016). Epi-1 also increases the amount of cytoskeletal protein and stabilizes the host cytoskeleton network by upregulating expression of proteins such as type II cytoskeletal keratin 8 and tropomyosin α-1 chain in zebrafish (Huang and Chen, 2013).

## Potential Application of Epinecidin-1 and Synthetic Epi-1

### Enhancing Fish Immunity in Aquaculture

#### Commercial Transgenic Fish

As domestication of orange spotted grouper as a food source prevailed, the infection of the fish by pathogens such as *V. vulnificus* has brought significant loss to the aquaculture industry and thus research for potential solution is extremely important (Pan et al., 2007). Introduction of epinecidin-1 gene into zebrafish can be used to create transgenic fish that are resistant to bacterial infection which is a major issue in aquaculture. The transgenic zebrafish was created by microinjection of [pTRL-M2.5 K.K.Epinecidin-1/DsRed plasmid DNA with transposase mRNA of pKJ/Tol2(A25)]. By utilizing an improved Tol2 transposon system, the transgenic fish has enhanced bacterial resistance against *V. vulnificus* and *Streptococcus agalactiae* and immunomodulatory effect was observed such as downregulation of IL-1β and TNF-α at 12 h post bacterial infection (Peng et al., 2010).

#### Aquaculture Feed

Epinecidin-1 can also be incorporated into commercial fish feed to enhance fish immunity. By using electroporation technique, plasmid encoding for epinecidin-1 was incorporated into *Artemia* cyst and was fed in combination with commercial fodder for 7, 14, 21 days resulting in a synergistic effect in protecting zebrafish against *V. vulnificus*. This method of enhancing fish defense against pathogens is much safer, cheaper, and only acts temporarily as it does not alter the genome of fish as compared to transgenic fish (Jheng et al., 2015).

The downside of the method mentioned above is that the epinecidin-1 gene cannot fully integrate into *Artemia* genome, thus transgenic *Artemia* using a Tol-2 transposon system with germ-line transmission of epinecidin-1 gene was created. A delayed death was observed in Nile tilapia fry fed with transgenic decapsulated *Artemia* cysts for 2 weeks prior to infection with *Streptococcus iniae* or *V. vulnificus* (204). The study demonstrated that feeding the tilapia fry with Epi-1 expressing *Artemia* cysts confers protection against bacterial infection through distinct immunomodulatory effects between *S. iniae* and *V. vulnificus* (Ting et al., 2018). In detail, there was no significant induction of *Tlr*-7/MyD88-mediated pathogenic response in transgenic cyst fed group as compared to the control group challenged with *S. iniae*. In addition, increased expression of IL-1β and IL-12 were observed in the transgenic cyst fed group, suggesting that the transgenic cysts enhance immunity of tilapia fry via pro-inflammatory response against Gram-positive bacterial infection. An anti-inflammatory response was also evident in transgenic cyst fed group with downregulated Cxcl-10 expression, suppressing potential acute inflammatory responses which can lead to cytokine storm and sepsis in tilapia fry. Conversely, *V. vulnificus* (204) induced *Tlr-5/MyD88/Traf-6-*mediated immune response in control tilapia fry while enhanced immunity via increased *Tlr-2* and *Tlr-5*-mediated immune response was observed in response to *V. vulnificus* (204) infection in transgenic cyst fed group. Overall, it is concluded that the Epi-1 expressing Artemia cysts enhance the tilapia fry immunity against bacterial infection (Ting et al., 2018).

On the other hand, epinecidin-1 can also be synthesized by *E. coli* protein expression system in which the recombinant BL21 *E. coli* harboring the epinecidin-1 peptide fusion dsRed protein in pET28a vector was used. *In vitro* study of the recombinant epinecdin-1 indicates the bactericidal effect among Gram negative strain including *E. coli, K. oxytoca, P. aeruginosa*, and *V. vulnificus* as well as Gram positive strain such as *Enterococcus faecalis. S. aureus* subsp. and *S. agalactiae* with MBC ranging from 300 to 350 μg/mL. Additionally, when different concentrations of recombinant epinecidin-1 BL21 *E. coli* was fed together with fish fodder to zebrafish and grouper for 30 days, the survival rates against *V. vulnificus* (204) was enhanced in a dose dependent manner along with immunomodulatory effect which can be observed with the increase in TNF-1 in grouper, TLR4, IL-1, NOS2, and NF-κB in zebrafish within 96 h post-infection. It was also speculated that epinecidin-1 is able to downregulate TNF-1 in grouper and antagonize TLR-4 binding thus reducing the TNF and IL-1β expression in zebrafish which enhance the survival. This method is regarded as a more time- and cost-effective method and is more practical for large scale production in aquaculture farms (Pan et al., 2012).

#### Gene Transfer

As there are concerns that transgenic fish may pose a risk to the native gene pools and natural ecosystems, the gene transfer by electroporation technique may serve as an alternative in creating fish with enhanced immunity. Studies were performed by injecting plasmid under CMV promoter which encodes for epinecidin-1 into the skeletal muscle of adult zebrafish (90 μg of DNA) or grouper (70 μg of DNA) followed by electroporation, and bactericidal effect was obtained against *V. vulnificus* challenge and lowering the level of inflammatory cytokines such as TNF-α and IL-1β upon bacterial infection which may involve in triggering sepsis. Thus, the plasmid encoding epinecidin-1 is able to protect fish against bacterial challenge and modulate the immune system (Lin et al., 2009a; Lee et al., 2013; Dunham and Winn, 2014).

### Topical Application for Human

Epi-1 is suitable for topical application as it is cytotoxic against bacteria, fungi, or parasites found on skin surface and has wound healing effect. Epi-1 destroyed membranes of *C. albicans* and had a MIC of 25 μg/mL. A study which added Epi-1 into New Touch cleaning solution, a vaginal cleaning solution with a pH of 4.2–4.6 and was stored for 7 and 14 days at 4 and 25°C showed that at 4°C storage temperature, an average of 2-fold inhibition on *C. albicans* was observed in Epi-1 combined with the New Touch Cleaning solution which performed better compared to other cleaning solutions with Epi-1. Thus, combination of Epi-1 with vaginal cleaning solution has better microbicidal activities and was more effective in preventing vaginal infection than the cleaning solution alone (Pan et al., 2009, 2010b).

The protozoan parasite, *T. vaginalis* and its metronidazole-resistant strain which often causes vaginal infection is also susceptible to Epi-1 in a dose and time dependent manner with experimental results showing that 60 min of treatment with 200 μg/mL for T1 strain and 25 μg/mL for ATCC 50143 were effective for genitourinary infection (Pan et al., 2009). In addition, a 400 μg Epi-1 treatment in mice had a 92–100% cure rate analyzed by wet mount, AO staining, and qPCR against the metronidazole-resistant strain which is similar efficacy as furazolidone (the current available treatment) and no toxicity was observed after a 2-weeks (1 mg/day) Epi-1 treatment which implies that Epi-1 is a potential microbicide against *T. vaginalis* infection (Huang et al., 2018a). Besides that, Epi-1 can be used as a as a component in cosmetic products due to the cytotoxic effect against *P. acnes*, thus making it effective in treating acne or surface lesions containing *P. acnes*. In addition to treating acne, the ability of Epi-1 to enhance keratinocyte proliferation and therefore cell renewal can accelerate the wound healing process, and when combined with collagen may be valuable to the cosmetic industry (Huang et al., 2013, 2017b).

### Synergy With Antibiotic

A few experiments have studied the synergistic activity of Epi-1 and antibiotics. Using checkerboard titration method, synergistic activity against MRSA was observed when Epi-1 was combined with streptomycin and kanamycin (Lin et al., 2013). Furthermore, using a turbidity fall assay, Epi-1 exhibited synergistic effect with amoxicillin, metronidazole, and clarithromycin (triple therapy) against the antibiotic resistant *H. pylori* by significantly lowering the MIC (Narayana et al., 2015). Thus, Epi-1 combination with antibiotics is more efficient than antibiotics alone and this is crucial to reduce drug dose and prevent bacteria resistance (Lin et al., 2013).

### Vaccine

Epi-1 was evaluated to serve as vaccine in place of using formalin-inactivated vaccine against JEV (Huang et al., 2011; Chen and Wu, 2013). This is because vaccines against JEV so far have been shown to have adverse effects. The test was done on 1-week-old C3H/HeN neonate mice and these were divided into groups. Each group was injected with either 1.5 × 10^7^ plaque forming units (pfu) of JEV alone, formalin-inactivated JEV vaccine (10 μg/mice), Epi-1-JEV-inactivated vaccine (10 μg/mice), or PBS alone. For Epi-1-JEV-inactivated vaccine, these were prepared by formulating a solution of Epi-1 (200 μg/mL) plus 50 × LD_50_ of JEV (1.5 × 10^7^ pfu). After this primary injection, two boosters were given on days 14 and 28. Serum was then collected for serological analysis and mouse survival was monitored daily for 53 days. Anti-JEV titers were measured by ELISA and the results showed poor activity of antibodies on the primary immunization in both vaccinated groups. After booster 1, both vaccinated mice showed increase in anti-JEV-E titer and the second boost increased the level of anti-JEV even more. Although each result showed that Epi-1-inactivated vaccine had higher anti-JEV-E titer than formalin-inactivated JEV vaccine. Moreover, with this challenge, the anti-JEV-titers increased significantly to 200 units on day four in both vaccinated groups. When the mice that survived were re-challenged with 50 × LD_50_ of JEV results showed that Epi-1-JEV immunized mice were all alive, whereas for formalin-JEV immunized mice, only one mouse died, and control group died within a week. Thus, Epi-1-based JEV-inactivated vaccine performed the same as or slightly better than the conventional vaccine in preventing JEV infection in neonate mice.

## Pharmacokinetics and Toxicity Studies of Epi-1

Pharmacokinetics involves the time course of a drugs presence in the body after drug administration and describes the processes such as absorption, distribution, metabolism, and excretion (ADME) of the drug. The association between drug concentration measured in body fluid such as blood, plasma, and urine together with therapeutic effects or/and toxicities of the drug is determined by pharmacokinetic principles (Miah et al., 2019). The concentration-time curve shows a decreasing curve after a single dose intravenous dose of 25 μg/fish of Epi-1 to male tilapia and the serum concentration was observed at 20, 30, 60, 120, and 180 min post injection. The maximum observed mean concentration for IV dosing of Epi-1 was 1342.13 ng/mL after 20 min with t_1/2_ of 60–80 min (Pan et al., 2007). The pharmacokinetics of different methods of administration were also studied by injecting 25 μg of Epi-1 into healthy Wistar rats IV, subcutaneously (SC), and intraperitoneally (IP). The result showed that IV injection shows the highest bioavailability of 15% while SC and IP had a bioavailability of 4% at 10 min after injection with steady decrease in Epi-1 serum concentration seen in SC and IP route. Thus, IV injection is the best method of administration for Epi-1 that resulted in the highest bioavailability among the three methods tested (Pan et al., 2014). Although a steady decrease of Epi-1 serum concentration is observed in SC and IP routes, the metabolism of Epi-1 such as into cleavage products in blood is still uncertain and thus more studies analyzing the pharmacokinetics of Epi-1 are needed (Pan et al., 2014).

Understanding the toxicity is crucial to ensure that the therapeutic agent does not cause any adverse effect at the working concentration. Haemolysis in humans can lead to the accumulation of hemoglobin in plasma and hemoglobinemia which subsequently leads to hemoglobinuria, ultimately causing clinical complications such as kidney damage and Fanconi syndrome. Thus, the AMPs used for systemic drug development must possess low toxicity against red blood cells (Misztal and Tomasiak, 2011; Oddo and Hansen, 2017). The haemolytic activity of AMP is higher when the peptide is more lipophilic and has a stable amphipathic secondary structure (Tossi et al., 2000). It should also be noted that tryptophan (W), lysine (K) and arginine (R) residues have the strongest effect on haemolytic of the AMP either through charge or hydrophobicity modulation (Oddo and Hansen, 2017). Generally, Epi-1 peptide has shown to exhibit low haemolytic activity but is, as expected, dose-dependent (Lin et al., 2013; Huang et al., 2018a). An *in vitro* study reported that the synthetic 25-mer C-terminal-amidated Epi-1 exhibits haemolytic activity on human erythrocytes at concentrations above 0.83 μM but not at 0.42 μM. Meanwhile, another study observed that the Epi-1 causes 100% haemolysis in human erythrocytes at 50 μg/mL (Yin et al., 2006; Pan et al., 2014). These studies indicate that the haemolytic activity of Epi-1 only occurs at high concentration. Moreover, Epi-1 does not cause nephrotoxicity as there is no observable cytotoxicity after treatment at concentration up to 10 μg/mL toward BHK21 cells (Su et al., 2017). This phenomenon was suggested by the fact that the epinecidin-1 sequence is a product of natural selection, thereby it has net positive charge to which it is electrostatically attracted to the anionic surface charge of bacterial cell membrane. Therefore, the electrically neutral eukaryotic membrane is not significantly affected by Epi-1 at low concentrations (Huang et al., 2018a). Through MTS assay, Epi-1 concentration at 50–800 μg/mL was added to grouper brain and spleen cells and incubated for 96 h to determine its cytotoxicity and result showed no toxicity (Wei et al., 2016). Furthermore, Grouper fin-1 (GF-1) and cured barramundi brain (cBB) cell lines were also tested and 4 μg/mL and 8 μg/mL of Epi-1 (CP643-1) were its maximal non-cytotoxic concentration, respectively (Chia et al., 2010).

*In vivo* study of Epi-1 in fish also showed no toxicity (Pan et al., 2007, 2011). A study utilizing intramuscular (IM) bolus injection of Epi-1 into mice showed no observed systemic toxicity even at high concentration (100 mg/kg), however eye narrowing was observed in one of three mice after 75 and 100 mg/kg Epi-1 administration but mostly recovered 5 h after treatment (Pan et al., 2014). Similarly, eye irritation test performed on rabbit (1 mg/eye/day for 7 days) showed lacrimation on day 2 and 3 but quickly recovered, the study also concluded that Epi-1 has no adverse toxicity when given by oral, dermal or ocular routes (Narayana et al., 2015). Although *in vivo* application on animal models may be safe, the application on human requires further research to determine the optimal therapeutic range of Epi-1 as high doses of Epi-1 may result in haemolysis.

## Limitations and Future Directions

Although Epi-1 exhibits promising pharmacological activities, there are potential challenges and limitations that may impede the clinical applications of Epi-1. These limitations include the physical instability of Epi-1 under physiological conditions, toxicity, the high cost of production and potential drug resistance.

Basically, Epi-1 is subjected to structural changes due to the possible alterations in *in vivo* conditions, including pH, temperature, and enzyme activity, thus affecting the specific three-dimensional structure of Epi-1 in conferring its beneficial biological activities. These factors may limit the parenteral use of Epi-1, whereby the antimicrobial activity of many antimicrobial peptides is reduced due to significantly lower concentrations in biological fluids such as plasma, serum, saliva, and sputum. The reduced antimicrobial activity may be caused by the high salt content in the body fluids which may interfere with the electrostatic interaction between the peptide and the surface of the bacteria (Batoni et al., 2011). Furthermore, Epi-1 may have low oral bioavailability due to the possibility of its rapid enzymatic degradation occurring along the gastrointestinal tracts. Similarly, subcutaneous and intraperitoneal injection of Epi-1 resulted in low bioavailability of 4.4% in mice (Pan et al., 2014). Generally, peptides is known to have short half-life after administration through intravenous injection (IV) due to the inactivation by proteolytic enzymes in the blood plasma and rapid clearance by the liver and kidney (Vlieghe et al., 2010; Mahlapuu et al., 2016). The t_1/2_ of Epi-1 was determined to be within 60–80 min after IV administration in fish and mice (Pan et al., 2007, 2014). The linear structure of the peptide may be potentially one of the factors causing it to be easily attacked by proteolytic enzymes in addition to its cationic properties and proteolytic susceptibility of a sequence with lysine residues (K) (Knappe et al., 2010; Kang et al., 2014). These discrepancies have been highlighter in comparisons between *in vitro* and *in vivo* efficacies of the peptides, implying that more *in vivo* studies should be done to provide a more accurate determination of the antimicrobial activity of Epi-1 in clinical settings (Mahlapuu et al., 2016). Today, protein modification techniques include the cyclization of peptide by linking C- and N-terminus, N-terminal acetylation or using D or non-natural amino acid are potential strategies that can be employed for the stability improvement of Epi-1 (Kang et al., 2014). Moreover, many nanoformulation techniques have also emerged to overcome these restraints to the use of such cationic AMP, such as Epi-1, hence improving the stability and delivery of these molecules (Zhang et al., 2012).

The hemolytic activity observed is one of the potential toxicities reported at high dose of Epi-1. Additionally, the broad spectrum antimicrobial activity of Epi-1 may not be beneficial for targeted therapies. An unselective AMP can eliminate both beneficial and pathogenic microbes in the host system, and may allow other invasive pathogens to enter the system. This is because indigenous microflora is important to human body in terms of acquiring nutrients and providing protecting against pathogens (Aoki et al., 2012; Kang et al., 2014). Hence, further effort can focus on the selectivity enhancement of Epi-1 to target specific pathogens, representing an effective approach for reducing toxicity. For instance, Epi-1 can be conjugated to a targeting domain which can specifically recognize certain pathogenic microorganisms and hence selectively eliminate the pathogens from the normal microflora in the host (Eckert et al., 2006; Kang et al., 2014). Given that only limited reports are available on the toxicity of Epi-1 in animal models, more research is needed to determine the optimal therapeutic dose for future human application in clinical trials (Yin et al., 2006).

Another limitation concerning the development of AMP is the high cost of large-scale manufacturing of the peptide (Ghosh et al., 2019). Hence, there is a growing need for a cheaper Epi-1 peptide production platform. One of the strategies to overcome the expensive production cost is to develop new expression systems, such as the use of *E-coli* expression system (Pan et al., 2012). Furthermore, instead of synthesizing the full epinecidin-1 sequence, a shorter sequence can be produced. A truncated peptide of epinecidin-1 named epinecidin-8 (EP-8) with the amino acid sequence of FIFHIIKGLFHAGKMI was synthesized and shown to retain the antibacterial activity and anticancer properties against HeLa and HT1080 cells of Epi-1. EP-8 even possesses a better antibacterial activity against *S. aureus* with a MIC of 6.25 μg/mL compared to Epi-1 with MIC of 50 μg/mL. Both Epi-1 and EP-8 also have synergistic activity with streptomycin or kanamycin against MRSA infection, thus having the potential of being developed as a more potent and cost-effective therapy by reducing the dosage of agent as well as decreasing the possible adverse reaction (Lin et al., 2013). Despite that, the production of peptides in bacteria is subjected to proteolytic degradation and the requirement for multiple purification steps of recombinant peptides is not cost-effective. Therefore, more research should explore on the development of cost-effective synthesis and purification of Epi-1, thereby enabling commercial enterprises to produce Epi-1 in large scale for future therapeutic applications in human.

Microbial pathogens strive to survive by colonizing and exploiting various tissues in a host, thus they are subjected to host defenses exerted by AMPs. Therefore, it is unrealistic that there is absence of any pathogens that resist AMPs (Yeaman and Yount, 2003). To be used as an anti-infective agent, it is imperative to address the potential emerging issue of Epi-1 resistance which will bring great implications on the future development of Epi-1 as a therapeutic agent to prevent or treat infection. Clearly, we have relatively limited understanding of how bacteria would acquire resistance to Epi-1. At time of writing, there is no study reporting on the mechanism of Epi-1 resistance developed by microbial pathogens as well as any exploratory study on potential development of Epi-1 resistance. With the current knowledge on AMP, it has known that drug resistance will inevitably emerge after a long exposure to AMP via several mechanisms, including modification of surface structures or reduction of surface affinities between AMP and cell membrane, the development of AMP efflux pump as well as the production of AMP-inactivating enzymes (Zhang et al., 2019). Nonetheless, the potential problem of acquired and cross-resistance to Epi-1 should be carefully monitored and taken into consideration anticipating the increasing implementation of Epi-1 in future clinical settings.

## Conclusion

Marine AMPs have huge potential to be developed for application in human and aquaculture as it is a natural antibiotic against a plethora of marine microbes. The present study reviewed the background information of epinecidin-1 including the peptide sequence, structure and expression as well as its toxicity and pharmacokinetics. Furthermore, this review also covers the potential pharmacological activities of epinecidin-1 presented by studies evaluating the uses of the synthesized 21 amino acid Ep-1 peptide derived from epinecidin-1 in various aspects, including antibacterial, antivirus, antifungal, immunomodulatory, anticancer, and wound healing properties. The potential application of the synthetic Epi-1 is also discussed. Studies on synthetic Epi-1 activities on various pathogens show that it does inhibit the pathogens mentioned above which means it may be effective for other pathogens as well hence, further experiments can be done so that it can be use therapeutically for other infections. Especially for fungal and yeast infection, more *in vivo* studies may be needed to further support its therapeutic effects. As mentioned earlier, the mechanism of action for viral inhibition and antibacterial effect is still inconclusive and more research is needed to understand how it acts. There are numerous potential applications of epinecidin-1 which includes gene transfer or transgenic organism development and the development into a therapeutic or prophylactic drug as an alternative to antibiotics, antibiotics adjuvant, vaccine adjuvant for human, and aquaculture usage. However, the nature of the epinecidin-1 including the characteristics, peptide sequence and processing enzymes and cleavage sites, mechanism of action, pharmacokinetics, pharmacodynamics, the potential toxicity, and optimal therapeutic dose must be further explored. Taken together, Epi-1 possesses immense pharmaceutical values, warranting further research and clinical studies to explore and validate its various therapeutic applications.

## Author Contributions

The writing was performed by PC, MM, EL, and LT. The manuscript was further critically reviewed and improved by W-LL, Y-WH, PP, LT, L-HL, and B-HG. Additionally, B-HG, LT, and K-GC provided vital guidance and insight to the work. The project was conceptualized by B-HG.

### Conflict of Interest

The authors declare that the research was conducted in the absence of any commercial or financial relationships that could be construed as a potential conflict of interest.
