# Supplementary material for: Epinecidin-1, an Antimicrobial Peptide Derived From Grouper (Epinephelus coioides): Pharmacological Activities and Applications
Source: Front Microbiol. 2019 Nov 20;10:2631. doi: 10.3389/fmicb.2019.02631 (PMC6879556; doi:10.3389/fmicb.2019.02631)
Supplement: Supplementary file 1 [file Table_1.DOCX]

**Title:** **Epinecidin-1, an antimicrobial peptide derived from Grouper (*Epinephelus coioides*): pharmacological activities and applications.**

Supplementary material


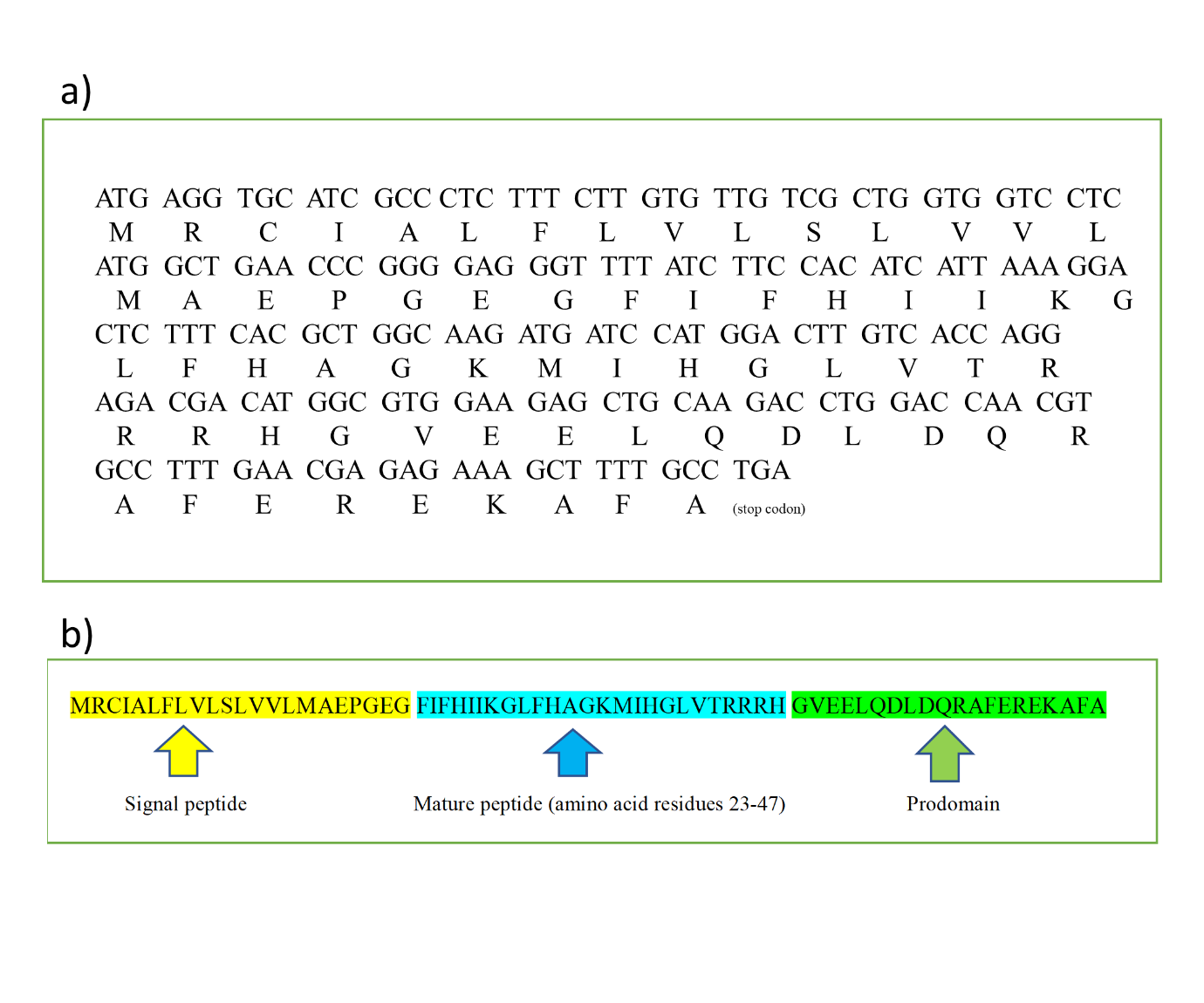


Figure S1. a) The 204 base pair open reading frame of epinecdin-1 and the respective amino acid residue based on Yin et.al (Yin et al., 2006) and GeneBank (accession no. AY294407.1). b) The predicted domains of signal peptide, mature peptide and prodomain of the 67 amino acid sequence epinecidin-1 prepropeptide (Yin et al., 2006).
